# Decoding sequence recognition code of nucleic acid‐binding proteins of human‐infecting DNA viruses

**DOI:** 10.1002/imt2.70170

**Published:** 2026-09-08

**Authors:** Meifang Tang, Shifei Yang, Haiqian Ma, Ruoying Guo, Jie Chen, Hui Zhang, Jinghan Zhang, Wei Sun, Yun Wei, Ligang Fan, Jian Yan

**Affiliations:** ^1^ Ministry of Education Key Laboratory of Resource Biology and Biotechnology in Western China; Shaanxi Provincial Key Laboratory of Biotechnology; School of Medicine Northwest University Xi'an China; ^2^ Tung Biomedical Sciences Centre, Department of Biomedical Sciences, College of Biomedicine City University of Hong Kong Hong Kong China; ^3^ Department of Urology The First Affiliated Hospital of Xi'an Jiaotong University Xi'an China; ^4^ Department of Precision Diagnostic and Therapeutic Technology The City University of Hong Kong Shenzhen Research Institute Shenzhen China

## Abstract

Human‐infecting DNA viruses remain major health threats, yet the DNA‐recognition mechanisms of their nucleic acid‐binding proteins (NBPs) are poorly understood. Here, we systematically profiled 103 viral NBPs from human‐infecting DNA viruses, with three NBPs from non‐human‐infecting DNA viruses as controls, using high‐throughput screening. This analysis identified diverse DNA‐binding motifs and specificity modules, including convergent recognition of a conserved CCACC motif across phylogenetically distant viruses. Notably, viral NBP binding‐site distributions varied with genome size, and several NBPs from small‐genome viruses showed enrichment on mitochondrial DNA. Functional assays further supported their mitochondrial association and effects on mitochondrial membrane potential. By integrating an ivTRT‐based ssDNA‐SELEX workflow, we further found that ssDNA viral NBPs recognize dimer‐like and inverted‐repeat sequences with potential to form stem‐loop structures. Collectively, this study constructs a comprehensive viral NBP DNA‐recognition atlas, offering a fundamental resource for elucidating viral genome recognition mechanisms, virus–mitochondria interactions, and developing future antiviral strategies.

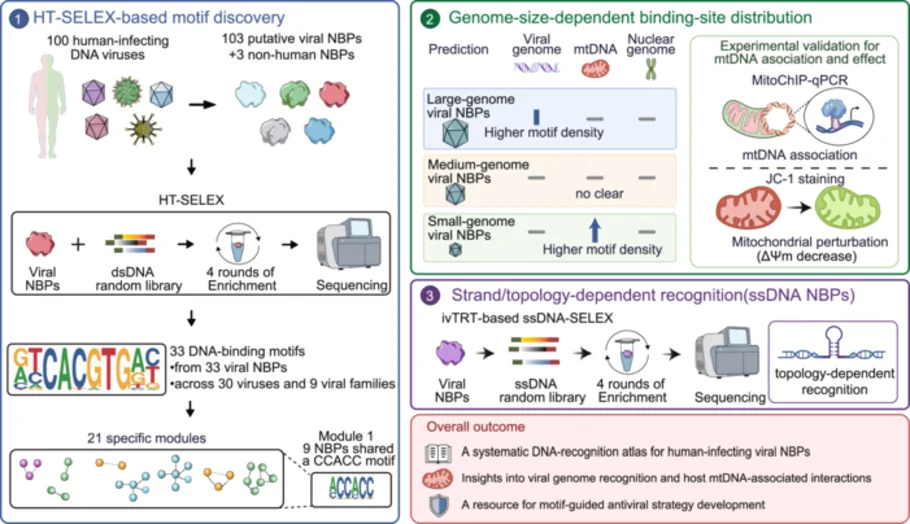

To the Editor,

Pathogenic DNA viruses have repeatedly caused widespread morbidity and mortality throughout history, posing a persistent threat to global public health. Hepatitis B virus (HBV) causes chronic liver disease and hepatocellular carcinoma [1], while recent mpox outbreaks highlight the continuing risk posed by emerging DNA viruses [2], underscoring the need for strategies targeting conserved viral mechanisms.

Mitigating this burden requires novel antivirals targeting proteins that govern key viral processes. Among these, viral nucleic acid‐binding proteins (NBPs), including capsid, nucleocapsid, and genome‐associated DNA‐binding proteins, interact with viral nucleic acids and collectively contribute to genome replication, viral gene expression, genome packaging, and virion assembly [3, 4]. Beyond the nuclear genome, mitochondrial DNA (mtDNA) represents another relevant host DNA compartment because its compact genome is comparable in scale to those of many small DNA viruses. Moreover, mitochondrial function is frequently perturbed during viral infection, and mitochondria play central roles in antiviral innate immunity [5, 6]. Although selective recognition of cognate viral DNA is important for viral genome packaging [4], viral NBPs have mostly been studied in individual viral systems [7, 8]. Their DNA‐binding specificities across diverse human‐infecting DNA viruses remain poorly characterized.

Here, using high‐throughput systematic evolution of ligands by exponential enrichment (HT‐SELEX) [9], we characterized 103 putative NBPs from 100 human‐infecting DNA viruses representing all known human DNA viral genera, with three non‐human‐infecting controls. Genome‐wide motif scanning revealed genome size‐associated differences in predicted binding‐site distributions, with several small‐genome viral NBPs showing increased mtDNA motif‐hit enrichment. By integrating an in vitro transcription–reverse transcription (ivTRT)‐based ssDNA‐SELEX workflow with HT‐SELEX, we further identified dimer‐like motifs, including inverted‐repeat patterns, in a subset of ssDNA virus‐derived NBPs, suggesting possible recognition of stem‐loop‐forming sequences. Overall, this study establishes a systematic DNA‐recognition atlas for viral NBPs and provides a valuable resource for investigating viral genome recognition, which is essential for developing new antiviral strategies.

## DNA‐BINDING SPECIFICITIES OF 33 VIRAL NBPS FROM 30 DNA VIRUSES

Human‐infecting DNA viruses were curated from the NCBI Virus database and Virus‐Host DB, excluding entries with ambiguous taxonomy or unclear genome‐type annotations. After manual curation, we retained 103 putative NBPs from 100 DNA viruses spanning 92 species, 43 genera, and 14 families, and three NBPs from non‐human‐infecting viruses as controls (YP_010782993.1, NP_671816.1, AVM80381.1) (Figure 1A, Figure S1 and Tables S1, S2). Following recombinant expression of these NBPs, four iterative rounds of HT‐SELEX yielded robust enrichment for 33 NBPs from 30 DNA viruses representing nine families (Figure 1A and Table S3). Each NBP's binding specificity was represented by a position weight matrix (PWM; Table S4). Motif lengths ranged from 8 to 18 bp and peaked at 10 bp (13/33; ~39.4%; Figure 1B), indicating that most viral NBPs recognize DNA sequences of constrained length.

**Figure 1:**
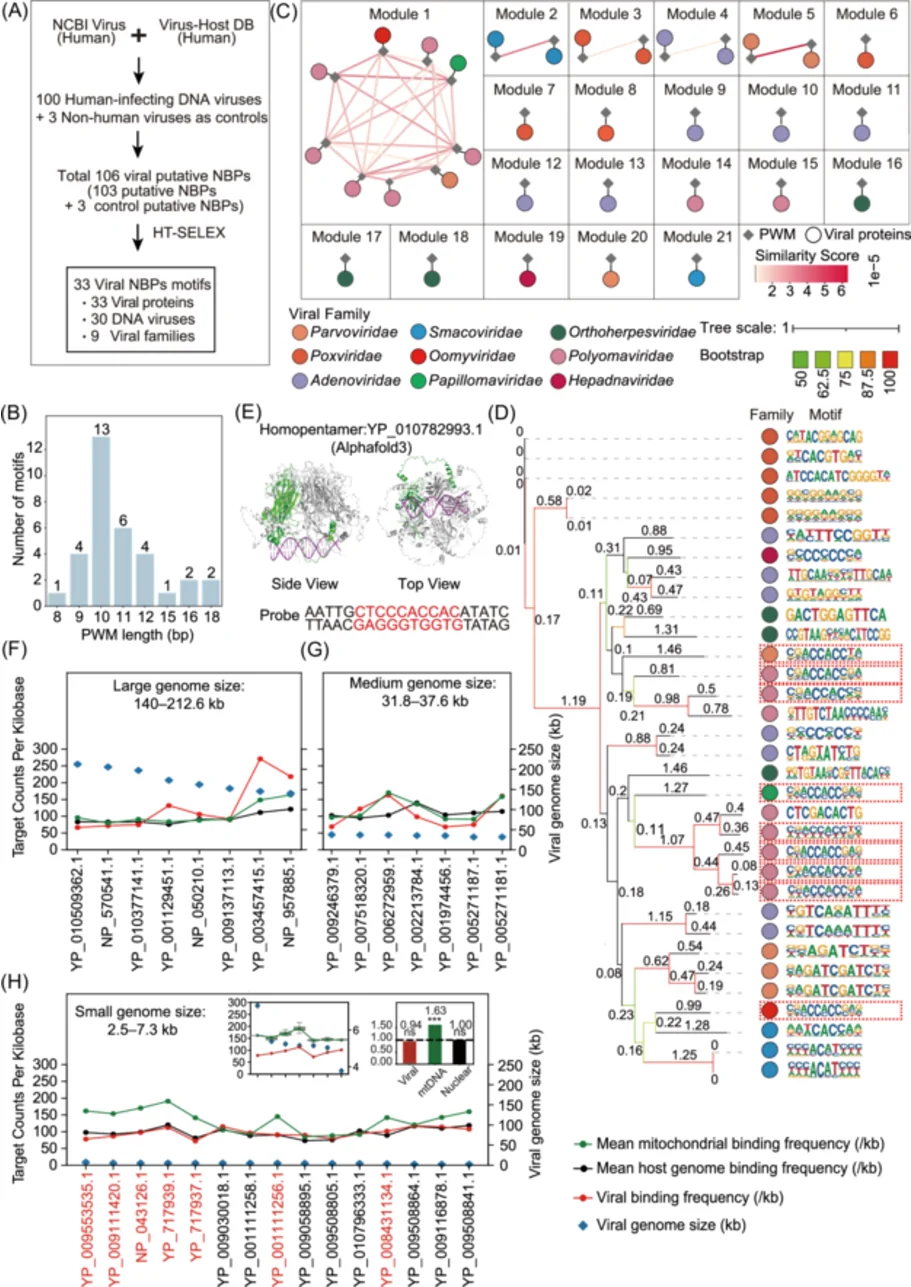
Screening and characterization of DNA‐binding motifs in viral nucleic acid‐binding proteins (NBPs) from human‐infecting DNA viruses. (A) Schematic overview of viral NBPs selection and the HT‐SELEX workflow. Three NBPs from non‐human‐infecting viruses (YP_010782993.1, NP_671816.1, AVM80381.1) were included as non‐human‐virus controls. Details are provided in Tables S1–S3. (B) Distribution of position weight matrix (PWM) model lengths for viral NBPs. Most PWM models were 10 bp in length. (C) Network of similarity among the obtained PWMs from viral proteins. Diamonds denote individual PWM models; circles denote viral proteins. Circle fill colors indicate the viral families from which the proteins originate. Dashed annotations label the protein IDs of viral proteins. An edge is shown between two nodes if the SSTAT similarity score > 1 × 10^−5^; edge colors reflect the SSTAT score. Details are provided in Table S5 and Figure S6. (D) Phylogenetic tree of 33 viral proteins from 30 viruses based on their amino acid sequences. Circle fill colors denote the corresponding viral families. Branch coloring reflects bootstrap support. The tree scale is substitutions per residue. Sequence logos show the corresponding DNA‐binding motifs. Proteins within module 1 are marked by the red dashed box. (E) AlphaFold3‐predicted model of the YP_010782993.1 pentamer bound to probe DNA, shown in top and side views, with one monomer highlighted in green. The protein structure is shown in gray, with the DNA probe (shown in purple) positioned to interact with the pentameric complex. The probe sequence is indicated below, with the CCACC motif highlighted in red. This binding site was predicted to interact with the viral genome and was validated by EMSA in Figure S5. (F–H) Distribution of predicted viral NBP‐binding sites across the human nuclear genome, human mtDNA, and cognate viral genomes. Viral NBPs were grouped by viral genome size relative to human mtDNA: (F) large genomes (~140–212.6 kb); (G) medium genomes (~31.8–37.6 kb); and (H) small genomes (~2.5–7.3 kb). The *x*‐axis shows viral protein IDs, the left *y*‐axis shows predicted target counts per kilobase (TCPK), and the right *y*‐axis shows viral genome size (kb). Green, black, and red circles indicate the mean predicted TCPK on human mtDNA, human nuclear chromosomes, and cognate viral genomes, respectively. Human mtDNA and nuclear chromosomes were analyzed using windows matched to each viral genome size. Blue diamonds indicate viral genome size. Red *x*‐axis labels denote the seven module 1 proteins from human‐infecting viruses that recognize the CCACC motif. The left inset highlights the distribution of their predicted mtDNA‐binding frequencies, and the right inset shows the three‐genomic‐context hypergeometric enrichment analysis of predicted NBP‐binding sites for these proteins. Significance levels are indicated as follows: ***, FDR‐adjusted *p* < 0.001. Details are provided in the Methods and Tables S8 and S9.

The reliability of the workflow was evaluated using two human transcription factors, ETV2 and HOXB13, as positive controls and an empty‐vector/tag‐only construct as background. Motifs recovered for ETV2 and HOXB13 in two independent experiments were consistent with their previously known motifs, whereas the background control did not yield any clear motif (Figure S2). In addition, motif‐containing sequences for the 33 motif‐positive viral NBPs generally showed progressive enrichment along SELEX cycles (Figure S3), and independent replicate HT‐SELEX experiments displayed highly consistent results (Figure S4). To validate the results, we performed electrophoretic mobility shift assays (EMSA) for 12 randomly selected NBPs representing both ssDNA and dsDNA viruses and distinct viral families (Figure S5). Together, these controls, replicate analyses, and orthogonal validation experiments support the reliability of the HT‐SELEX‐derived motifs.

## DIVERGENT AND CONVERGENT DNA‐BINDING SPECIFICITIES ACROSS VIRAL LINEAGES

Pairwise PWM similarity among the 33 motifs grouped the NBPs into 21 modules (Figure 1C, Figure S6 and Table S5). Sixteen modules (~76.2%) showed one‐to‐one NBP‐motif correspondence, indicating substantial inter‐lineage divergence. The remaining five revealed two evolutionary patterns. Modules 2–5 exemplify intra‐family conservation: pairs of NBPs from *Smacoviridae*, *Poxviridae*, *Adenoviridae*, and *Parvoviridae* share family‐specific motifs, consistent with a modular mode of viral evolution in which virion‐associated proteins may be inherited from common ancestral modules [10]. Strikingly, module 1 revealed inter‐family convergence: nine NBPs from four phylogenetically distant families, including *Parvoviridae* (*n* = 1), *Oomyviridae* (*n* = 1), *Papillomaviridae* (*n* = 1), and *Polyomaviridae* (*n* = 6), shared a core CCACC consensus motif, implying convergence at the level of DNA sequence recognition.

Based on amino acid sequences of all 33 viral NBPs, we constructed a phylogenetic tree (Figure 1D), which confirmed that modules 2–5 cluster within their viral family, whereas the nine NBPs in module 1 segregated into distinct clades, suggesting convergence independent of primary protein sequence homology. Since viral proteins exhibited structural similarity despite sequence divergence [10], we examined the predicted structures of module 1. Only YP_717939.1 from *Polyomaviridae* has an experimentally determined crystal structure [11], revealing a homo‐pentameric assembly symmetrically arranged around a central pore, with each subunit adopting a β‐sandwich jelly‐roll fold, a hallmark of viral capsid proteins [12] (Figure S7A). AlphaFold3 predictions showed that most high‐confidence models from dsDNA viruses adopted pentameric architectures broadly resembling YP_717939.1 (Figure S7 and Tables S6, S7). The two ssDNA virus‐derived module 1 NBPs, however, differed from this predominant structural pattern and showed lower global structural similarity to YP_717939.1 (TM‐score < 0.5): AVM80381.1 differed at both the subunit and assembly levels (Figure S7F), whereas YP_008431134.1 retained pentameric symmetry despite divergent per‐subunit folds (Figure S7G). These observations suggest that, although most module 1 NBPs recognize CCACC‐containing motifs, this motif‐level convergence is implemented through divergent structural mechanisms rather than a single shared binding mode. To explore the structural basis of CCACC motif recognition, an AlphaFold3 model of the YP_010782993.1‐DNA complex reveals that DNA threads through a central pore within the pentamer, with the upper subunit surfaces forming cooperative contact points (Figure 1E).

## GENOME SIZE‐ASSOCIATED BINDING PATTERNS OF VIRAL NBPS

To compare the overall abundance and distribution of potential motif‐matching sequences across different genomic contexts, we applied the viral NBP PWMs to scan their cognate viral genomes, size‐matched windows of the human nuclear genome, and mtDNA, quantifying normalized fold enrichment with one‐sided hypergeometric tests (fold enrichment > 1 and FDR‐adjusted *p* < 0.05). This analysis provided a group‐level view of predicted binding‐site density across viral, nuclear, and mitochondrial genomic backgrounds. Stratifying NBPs by viral genome size relative to mtDNA revealed three patterns across multiple *p* value thresholds (Figure S8 and Tables S8, S9). NBPs from large‐genome viruses (~140–212.6 kb) showed consistent enrichment on their cognate viral genomes across multiple thresholds (fold enrichment ≈ 1.31–1.39, FDR‐adjusted *p* < 0.001 for all comparisons; Figure 1F, Figure S8 and Tables S8, S9), a pattern that may reflect a higher density of motif‐matching sequences in their cognate viral genomes and may be consistent with preferential recognition of their own genomes over host genomic contexts. Medium‐genome NBPs (~31.8–37.6 kb) showed weaker enrichment, without a uniformly dominant genomic context across all thresholds (Figure 1G, Figure S8 and Tables S8, S9). In contrast, small‐genome NBPs (~2.5–7.3 kb) displayed the most consistent mtDNA enrichment, with fold enrichment increasing from ~1.35 (*p* < 0.05) to ~1.77 (*p* < 0.001) as stringency tightened; importantly, this association remained significant across all tested thresholds, confirming the robustness of the mitochondrial bias for this class of viral NBPs (Figure S8 and Table S8). This pattern was particularly evident among module 1 NBPs (Figure 1H). These results indicate a genome‐size‐associated redistribution of predicted motif‐site density across viral, nuclear, and mitochondrial genomic contexts.

To more stringently evaluate the mtDNA‐associated pattern, we constructed two complementary empirical nuclear‐genome null models using windows equal in size to human mtDNA. In the first analysis, motif‐hit counts on mtDNA were compared with those in randomly sampled nuclear windows. In the second, mutually non‐overlapping nuclear windows were selected according to their similarity to mtDNA dinucleotide composition. Across multiple FIMO thresholds, small‐genome viral NBPs generally showed higher mtDNA motif‐hit enrichment relative to both nuclear‐window null distributions, with the strongest and most consistent pattern observed among module 1 proteins, although composition matching reduced the magnitude of enrichment for some proteins (Figure S9 and Tables S10, S11). We further generated 1000 matched shuffled PWMs for each protein by permuting the A/C/G/T probability labels within each position, thereby preserving motif width and the per‐position probability‐value set while randomizing nucleotide identity. The original PWMs from small‐genome NBPs, particularly module 1 PWMs, generally produced more hits on mtDNA than their corresponding shuffled‐PWM controls across the four FIMO thresholds (Figure S10 and Table S12). This phenomenon may be related to the fact that human mtDNA is substantially larger than these small viral genomes and is therefore unlikely to be mistakenly recognized as a packaging substrate by NBPs. In addition, no comparable enrichment was observed for the non‐human‐virus control NBPs (Figure S11). The observed enrichment may instead reflect potential interactions with mtDNA or mtDNA‐associated pathways, with possible implications for mitochondria‐mediated antiviral innate immunity. Within each cognate viral genome, predicted NBP‐binding sites showed discrete, non‐uniform distributions with localized hotspots (Figure S12, Figure S13), resembling the organization of packaging signals in which multiple distributed recognition sites contribute to efficient genome encapsidation [13].

Focusing on module 1 NBPs, all human‐infecting members shared remarkably similar enrichment profiles across the full mtDNA, a pattern not observed for other NBPs (Figure S14A). Predicted sites showed pronounced enrichment in the noncoding d‐loop region (positions 16,024–576), with the highest level of gene densities observed at MT‐TS1, MT‐ND6, MT‐ATP8, and MT‐ND1 (Figure S14B,C and Table S13). To validate these predicted mtDNA‐enriched loci in human cells, we performed MitoChIP‐qPCR for five selected small‐genome viral NBPs (four module 1 and one non‐module 1), together with YP_009508805.1, a comparison viral protein with low predicted mtDNA‐binding potential. The five candidate NBPs showed significantly greater enrichment at the predicted mtDNA loci than the empty‐vector and the control protein (Figure 2A and Table S14). Quantitative PCR confirmed that all NBPs and YP_009508805.1 were efficiently overexpressed relative to the empty vector, excluding the failed expression for the low background (Figure S15 and Table S15).

**Figure 2:**
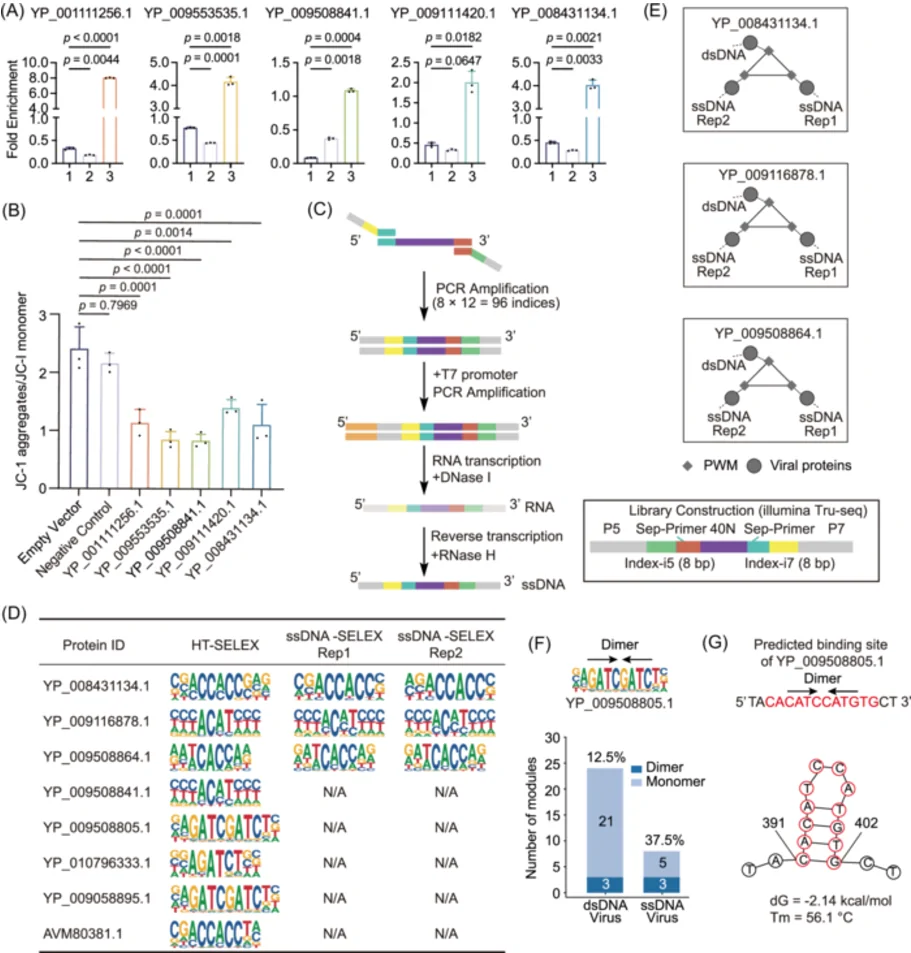
Comparative HT‐SELEX and ssDNA‐SELEX analyses reveal distinct DNA‐binding modes of ssDNA virus‐derived proteins. (A) Validation of viral NBP binding to mitochondrial DNA by MitoChIP‐qPCR. Group 1 = empty vector control; Group 2 = negative control protein; Group 3 = corresponding FLAG‐tagged viral NBP protein indicated atop each panel. MitoChIP‐qPCR detected mtDNA enrichment at 48 h post‐transfection. All viral NBPs showed significantly stronger mtDNA binding than the empty vector control, whereas the negative control had negligible enrichment. Data are shown as mean ± SD (*n* = 3 biological replicates). Statistics: Brown‐Forsythe and Welch ANOVA with Dunnett's correction for multiple comparisons. (B) Assessment of mitochondrial membrane potential (ΔΨm) in HEK293T cells by JC‐1 staining. HEK293T cells were transfected with plasmids encoding viral NBPs, an empty vector, or a negative control protein. Mitochondrial membrane potential was measured by JC‐1 staining coupled with flow cytometry. The fluorescence ratio of JC‐1 aggregates to monomers serves as a quantitative indicator of mitochondrial polarization, with a decreased ratio indicating depolarization. Data are presented as mean ± SD from three independent biological replicates. Statistical analysis was performed using one‐way ANOVA followed by Dunnett's multiple comparisons test against the empty vector group. (C) Schematic overview of the ssDNA library generation strategy for ssDNA‐SELEX. Color coding indicates defined sequence elements throughout the workflow, including primer regions, the 40‐nt randomized region, the T7 promoter, and the final Illumina Tru‐seq adapter‐compatible architecture. Primer sequences are listed in Table S3. (D) Comparative summary of motifs identified for ssDNA virus‐derived proteins using HT‐SELEX and ssDNA‐SELEX. For proteins tested by ssDNA‐SELEX, two independent ssDNA‐SELEX replicates are shown, demonstrating motif reproducibility across replicates. N/A indicates that no clear and reproducible motif was detected by ssDNA‐SELEX. (E) PWM similarity networks for three ssDNA virus‐derived proteins that yielded comparable motifs across HT‐SELEX and two independent ssDNA‐SELEX replicates. For YP_008431134.1, the HT‐SELEX motif was truncated to the first nine positions and compared with the 9‐bp ssDNA‐SELEX motifs to assess core‐sequence similarity. Diamonds represent individual PWM models, and circles represent viral proteins. Viral protein nodes are linked to their corresponding PWM models, and PWM models are connected when their SSTAT similarity score exceeds 1 × 10^−5^. Additional details are provided in Tables S4 and S18. (F) Classification of viral DNA‐binding modes across proteins derived from different viral genome types. Binding modes were categorized as monomeric motifs, corresponding to non‐repetitive binding sites, or dimeric motifs, characterized by two similar subsequences within the motif. The motif recognized by YP_009508805.1 is shown as an example of a dimeric binding mode. (G) Predicted secondary structure and genomic location of the YP_009508805.1 binding site in its viral genome. The predicted binding site can form a stem‐loop structure in which the dimeric motif contributes to the duplex stem region. The structure was modeled using mfold under conditions approximating human physiological temperature and ionic strength. Δ*G* indicates the predicted thermodynamic stability of the structure, and Tm indicates the predicted melting temperature.

Additionally, analysis of the mitochondrial‐to‐nuclear DNA ratio (Mt/N) showed that the YP_001111256.1 MitoChIP group exhibited a markedly elevated Mt/N ratio (~1050) compared with the mitochondrial input (~40–50), corresponding to an approximately 20‐fold enrichment and further supporting preferential mtDNA binding (Figure S16 and Table S16). Consequently, cells overexpressing these five NBPs displayed significantly reduced aggregate‐to‐monomer ratios relative to control cells transfected with YP_009508805.1 or empty vector, as measured by JC‐1 staining, indicating altered mitochondrial membrane potential (Figure 2B and Table S17). Together, these findings support a protein‐dependent association of the selected small‐genome viral NBPs with mtDNA enrichment and mitochondrial perturbation, although they do not establish a direct causal relationship.

## SSDNA‐SELEX REVEALS DISTINCT DNA‐BINDING MODES OF NBPS IN SSDNA VIRUS

To determine whether NBPs from ssDNA viruses exhibit strand‐state‐specific binding preferences preference, we developed an ssDNA‐SELEX method integrating strand‐specific ssDNA random libraries with iterative SELEX selection (Figure 2C, Figure S17A). To avoid common ssDNA‐generation methods that may produce mixed ssDNA/dsDNA products, in addition to dependence on chemically modified primers, physical strand separation, and tedious purification [14, 15], we established an ivTRT strategy. In this workflow, T7 promoter‐mediated transcription of synthesized DNA templates containing a 40‐bp region produced RNA libraries of random sequences [16]. Next, DNase I digestion removed template DNA, followed by reverse transcription to generate RNA–cDNA hybrids. RNase H digestion ultimately yielded ssDNA libraries as SELEX input, which was confirmed by gel electrophoresis with the expected fragment size (Figure S17B). Next‐Generation sequencing showed broad k‐mer coverage and normalized Shannon entropy close to 1 across *k* = 2–12, comparable to those of a depth‐matched simulated random reference, supporting the high complexity and near‐random composition of the input library (Figure S17C,D). To validate the method, we performed ssDNA‐SELEX on translocated in liposarcoma/fused in sarcoma (FUS/TLS), a protein with established ssDNA‐binding activity [17], supporting the reliability of the ssDNA‐SELEX method (Figure S17E).

Using this method, we profiled eight NBPs from ssDNA viruses and compared ssDNA binding motifs to those from HT‐SELEX (dsDNA motifs). Three proteins produced highly similar motifs between both methods (Figure 2D,E, and Table S18), indicating that their core sequence recognition is largely independent of DNA strand state. EMSA validated concentration‐dependent binding of two of these proteins to motif‐containing dsDNA probes (Figure S5). We subsequently examined whether systematic differences in binding specificity and motif structure could be observed across all NBPs, including those derived from ssDNA viruses that produced motifs only in HT‐SELEX but not in ssDNA‐SELEX. Across all 33 NBP motifs, the internal‐repeat classification identified two PWM binding patterns: non‐repetitive sequences (monomer‐like) and repetitive sequences containing two similar sub‐sequences (dimer‐like) (Figure 2F, Figure S18 and Table S19). Dimer‐like patterns were identified in 3 out of 8 ssDNA‐virus‐derived PWMs (37.5%) and 3 out of 24 dsDNA‐virus‐derived PWMs (12.5%), respectively. As a representative example, the motif recognized by the *Parvoviridae* protein YP_009508805.1 contains a pair of inverted repeats; sequence‐based secondary structure prediction suggests that the corresponding ssDNA region can fold into a stem‐loop, with the inverted repeats forming the duplex stem (Figure 2G). This increased prevalence of dimer‐like patterns in ssDNA‐virus‐derived PWMs may be related to the replication strategy of some ssDNA viruses, in which incoming single‐stranded genomes are rapidly converted to dsDNA replicative‐form intermediates that serve as templates for transcription and replication [18]. Given that Adeno‐associated virus (AAV; *Parvoviridae*) harbors terminal palindromes (inverted terminal repeats) required for replication and packaging [19], such dimer‐like motifs may reflect sequence architectures compatible with genome recognition across dynamic conformational states during the viral life cycle; however, given the limited sampling, this interpretation requires validation in larger datasets and direct experiments.

In conclusion, our systematic profiling of viral NBPs reveals both convergent and divergent DNA‐recognition principles across diverse human‐infecting DNA viruses. A shared CCACC motif is convergently recognized by nine NBPs from four distantly related viral families, with the dsDNA virus‐derived NBPs sharing a pentameric jelly‐roll scaffold, whereas the ssDNA virus‐derived NBPs adopt distinct architectures. We also uncover genome‐size‐dependent binding patterns, with small‐genome viral NBPs preferentially enriching at mtDNA and associating with mitochondrial perturbation, though causality awaits direct validation.

Several limitations should be considered when interpreting these findings. Failure to identify a clear sequence‐specific motif in HT‐SELEX does not necessarily conclude the absence of nucleic acid‐binding activity. Some viral NBPs may bind nucleic acids in a sequence‐independent and cooperative manner. In addition, the AlphaFold3‐based structural analyses are predictive and require experimental validation to confirm the inferred structural relationships. Although we showed that several small‐genome viral NBPs exhibited increased mtDNA motif‐hit enrichment and altered mitochondrial membrane potential, they do not establish a direct causal relationship between mtDNA binding and mitochondrial perturbation. Functional validation in infection models, along with mtDNA‐dependency assessment using ρ^0^ cells and direct examination of NBP–mtDNA co‐localization during viral infection, will be essential to establish the biological relevance of these interactions.

## AUTHOR CONTRIBUTIONS

**Meifang Tang**: Software; methodology; data curation; investigation; writing—original draft; writing—review and editing; visualization. **Shifei Yang**: Methodology; data curation; investigation; validation; visualization; writing—original draft; writing—review and editing. **Haiqian Ma**: Methodology; data curation; investigation; validation; writing—original draft; writing—review and editing. **Ruoying Guo**: Methodology; data curation; investigation; validation. **Jie Chen**: Software; data curation. **Hui Zhang**: Data curation; methodology; investigation; validation. **Jinghan Zhang**: Methodology; data curation; investigation; validation. **Wei Sun**: Software; methodology; visualization. **Yun Wei**: Methodology; data curation; investigation; validation. **Ligang Fan**: Conceptualization; methodology; data curation; investigation; validation; supervision; funding acquisition; visualization; writing—original draft; writing—review and editing; project administration. **Jian Yan**: Conceptualization; funding acquisition; writing—original draft; writing—review and editing; visualization; methodology; supervision; project administration.

## CONFLICT OF INTEREST STATEMENT

The authors declare no conflicts of interest.

## ETHICS STATEMENT

No animals or humans were involved in this study.

## Supporting information


**Figure S1:** SDS‐PAGE and anti‐FLAG Western blot analysis of all 106 purified viral candidate NBPs.
**Figure S2:** Positive and background controls for the HT‐SELEX workflow.
**Figure S3:** Round‐by‐round enrichment of motif‐containing sequences during HT‐SELEX.
**Figure S4:** Motif reproducibility between replicate HT‐SELEX experiments.
**Figure S5:** EMSA verification of SELEX‐identified DNA binding motifs for viral NBPs.
**Figure S6:** Network of similarity among the obtained PWMs from viral proteins.
**Figure S7:** Structural analysis of CCACC motif viral NBPs in module 1.
**Figure S8:** Three‐genomic‐context hypergeometric enrichment analysis of predicted NBP‐binding sites.
**Figure S9:** Empirical nuclear‐window null analyses of mtDNA motif‐hit enrichment.
**Figure S10:** mtDNA motif‐hit enrichment relative to shuffled‐PWM controls.
**Figure S11:** Genome‐wide binding analysis of non‐human‐infecting viral proteins.
**Figure S12:** Genome‐wide distribution of predicted binding sites for viral proteins in module 1 across viral genomes.
**Figure S13:** Genome‐wide distribution of predicted binding sites for viral proteins from modules other than module 1 across their cognate viral genomes.
**Figure S14:** Predicted binding landscape of module 1 viral NBPs from human‐infecting viruses across human mtDNA.
**Figure S15:** Validation of Flag‐NBP overexpression efficiency by quantitative PCR.
**Figure S16:** Mt/N ratio verifies mitochondrial purity and MitoChIP enrichment efficiency.
**Figure S17:** Quality assessment and validation of the ssDNA‐SELEX workflow.
**Figure S18:** Viral NBP PWMs classified as dimer‐like binding patterns containing two similar subsequences.

**Table S1:** Summary of viral information.
**Table S2:** Viral NBPs purification and motif summary.
**Table S3:** Primer sequences and probe sequences used in this study.
**Table S4:** PWM model generated from HT‐SELEX or ssDNA‐SELEX.
**Table S5:** SSTAT similarity scores among HT‐SELEX‐derived PWM models for 33 viral proteins.
**Table S6:** pLDDT Scores.
**Table S7:** Structural similarity scores between viral proteins.
**Table S8:** Three‐context group‐pooled hypergeometric enrichment across four FIMO *p* value thresholds.
**Table S9:** Predicted NBP‐binding sites and density‐based enrichment comparison across viral and host genomic backgrounds.
**Table S10:** Per‐PWM enrichment statistics for the genome‐wide random mtDNA‐length nuclear‐window null.
**Table S11:** Per‐PWM enrichment statistics for the dinucleotide‐matched non‐overlapping nuclear‐window null.
**Table S12:** Per‐protein mtDNA motif‐match statistics for original and matched base‐label‐shuffled viral NBP PWMs.
**Table S13:** Predicted binding sites of PWMs across human full‐length mitochondrial genomes.
**Table S14:** Statistical summary of MitoChIP‐qPCR analysis.
**Table S15:** Statistical summary of overexpression qPCR analysis.
**Table S16:** Statistical summary of mtDNA/nDNA ratio analysis.
**Table S17:** Statistical summary of JC‐1 assay analysis.
**Table S18:** SSTAT similarity scores among HT‐SELEX‐ and ssDNA‐SELEX‐derived PWM models for three ssDNA virus‐derived proteins.
**Table S19:** Internal‐repeat classification of 33 viral protein PWMs.

## Data Availability

Sequencing data generated in this study are available in the Gene Expression Omnibus (GEO) under accession numbers GSE309420 (https://www.ncbi.nlm.nih.gov/geo/query/acc.cgi?acc=GSE309420) and GSE335776 (https://www.ncbi.nlm.nih.gov/geo/query/acc.cgi?acc=GSE335776). The scripts used are available on GitHub (https://github.com/tang176-code/Virus). Supplementary materials (figures, methods, tables, graphical abstract, slides, videos, Chinese translated version, and updated materials) may be found in the online DOI or iMeta Science (http://www.imeta.science).
