# Supplementary material for: Decoding sequence recognition code of nucleic acid‐binding proteins of human‐infecting DNA viruses
Source: Imeta. 2026 Sep 8;5(5):e70170. doi: 10.1002/imt2.70170 (PMC13550974; doi:10.1002/imt2.70170)
Supplement: Supplementary file 1 [file IMT2-5-e70170-s002.docx]

**Supporting information to**

**Decoding Sequence Recognition Code of** **Nucleic Acid-Binding Proteins of Human-Infecting DNA Viruses**

**Running title:** DNA Recognition by Viral Nucleic Acid-Binding Proteins

Meifang Tang^1,2#^, Shifei Yang^1,3#^, Haiqian Ma^1#^, Ruoying Guo^1^, Jie Chen^2^, Hui Zhang^1^, Jinghan Zhang^1^, Wei Sun^1^, Yun Wei^1^, Ligang Fan^1^*, Jian Yan^1,2,3,4^*

^1^ Ministry of Education Key Laboratory of Resource Biology and Biotechnology in Western China; Shaanxi Provincial Key Laboratory of Biotechnology; School of Medicine, Northwest University, Xi’an 710069, China.

^2^ Tung Biomedical Sciences Centre, Department of Biomedical Sciences, College of Biomedicine, City University of Hong Kong, Hong Kong, China.

^3^ Department of Urology, The First Affiliated Hospital of Xi’an Jiaotong University, Xi’an 710061, China.

^4^ Department of Precision Diagnostic and Therapeutic Technology, The City University of Hong Kong Shenzhen Research Institute, Shenzhen 518057, China.

^#^ These authors contributed equally: Meifang Tang, Shifei Yang, Haiqian Ma

* Correspondence: Ligang Fan (fanlg@nwu.edu.cn), Jian Yan ([jian.yan@cityu.edu.hk](mailto:jian.yan@cityu.edu.hk)).

**METHODS**

**Selection of human-infecting DNA Viruses and identification of putative viral NBPs**

To systematically characterize the DNA-binding specificities of viral putative NBPs from human-infecting DNA viruses, we curated a comprehensive virus set from public databases. Human-infecting DNA viruses were initially collected from the NCBI Virus database [1] and Virus-Host DB [2] (retrieved on 2023-10-17). In NCBI Virus, viruses were retrieved using the filter “Host = Homo sapiens (taxid:9606),” whereas in Virus-Host DB, the “Human viruses” dataset was selected. The two datasets were harmonized using NCBI RefSeq complete-genome accessions (NC_ accessions) as common identifiers. These searches yielded 867 and 925 viral accessions, respectively, corresponding to 1,792 accession records in total. After merging the two datasets and removing duplicate accessions, 1,130 unique viral accession records were retained, including 662 shared between the two databases, 263 unique to Virus-Host DB, and 205 unique to NCBI Virus. Among these 1,130 unique viral accessions, 807 non-DNA viruses were excluded, leaving 323 preliminary candidate DNA viruses. These candidates were subsequently subjected to manual curation. A total of 223 viruses were removed according to the following criteria: 47 entries with ambiguous taxonomic assignments; 163 redundant viruses within the selected taxonomic groups; 2 viruses with RT-DNA genome types were removed and 11 viruses for which no identifiable putative nucleic acid-binding proteins could be identified. After these filtering steps, a final set of 100 human-infecting DNA viruses, representing 92 species, 43 genera, and 14 families, was retained for subsequent identification and screening of putative viral NBPs.

From these viruses, 103 putative viral NBPs were identified for downstream analyses. To ensure the rationality and reliability of the candidate library, all viral proteins were strictly selected based on predefined evidence for nucleic-acid-binding potential. A protein was retained as a putative viral NBP if its nucleic-acid-binding capacity was supported by at least one of the following three evidence categories: (i) annotated nucleic-acid-binding function or conserved nucleic-acid-binding domains documented in public databases, including NCBI, UniProt, and InterPro; (ii) direct experimental evidence of nucleic-acid-binding activity reported in peer-reviewed studies; or (iii) significant sequence homology or structural similarity to functionally validated viral NBPs with experimentally characterized nucleic-acid-binding properties. Proteins lacking support from all three categories were excluded from subsequent screening and functional validation. This standardized framework ensured the rationality and reliability of the candidate protein library for downstream characterization of nucleic-acid-binding preferences. In addition, three NBPs (YP_010782993.1, NP_671816.1, and AVM80381.1) derived from three viral species whose hosts are not humans were included as non-human-virus controls. The Marmota monax genome assembly (GCF_021218885.2) and additional viral sequences were retrieved from NCBI (GenBank/RefSeq). The human (hg38) and rat (rn7) genomes were used as reference sequences in this study. For additional details, see Table S1.

**Cloning, expression, and purification of viral NBPs**

The genes encoding the identified viral NBPs were cloned into the pET28a vector (which contains a 6 × His tag for protein purification) using standard molecular cloning techniques. The recombinant plasmids were then transformed into *Escherichia coli BL21(DE3)* for protein expression. Protein expression was induced with 1 mM IPTG for approximately 17 h at 17 °C. After expression, the cells were harvested at 8,000 rpm. The viral NBPs were purified from the cell lysate using nickel-nitrilotriacetic acid (Ni-NTA) affinity chromatography, following the manufacturer's instructions. The purity of the purified proteins was checked using sodium dodecyl sulfate-polyacrylamide gel electrophoresis (SDS-PAGE).

**Construction of the DNA input library for HT-SELEX and ssDNA-SELEX**

For the dsDNA input library, a randomized dsDNA input library with a 40 bp fully degenerate region (N40) was synthesized as the starting pool. Indexed sequences were incorporated via PCR using paired Illumina-type primers. Each 100 μL reaction contained **2 × Phanta Max Master Mix (Vazyme, P515-03)**, 10 μL each of 10 μM N5XX and N7XX primers, and 65 ng of DNA template in nuclease-free water. Amplification was carried out with an initial denaturation at 95 °C for 3 min, followed by 10 cycles of 95 °C for 15 s, 55 °C for 15 s, and 72 °C for 30 s, and finalized with 72 °C for 5 min before holding at 4°C. PCR products were analyzed by agarose gel electrophoresis, and fragments of the expected size were excised and purified using the **Monarch® DNA Gel Extraction Kit (NEB, #T1020L).** High-throughput sequencing was performed to evaluate nucleotide distribution, and only libraries exhibiting unbiased base frequency across the N40 region were retained for HT-SELEX [3–6].

For the ssDNA input library, the dsDNA library was reamplified to append a T7 promoter sequence in a 25 μL reaction containing 12.5 μL **2 × Phanta Max Master Mix (Vazyme, P515-03)**, 1 μL each of 10 μM Illumina-T7-F and Illumina-SELEX-R primers, and 3 μL dsDNA template. PCR cycling was conducted as follows: 95 °C for 3 min; 18 cycles of 95 °C for 15 s, 55 °C for 15 s, and 72 °C for 30 s; followed by 72 °C for 5 min and hold at 4 °C. RNA transcription was subsequently performed using the **T7 High Yield RNA Transcription Kit (Vazyme, TR101-02)** in a 20 μL reaction containing 2 μL 10 × transcription buffer, 0.4 μL each of ATP, CTP, GTP, and UTP, 5 μL of T7-promoter-modified DNA, 0.5 μL RNase inhibitor, and 2 μL T7 RNA polymerase mix. Samples were incubated at 37 °C for 16 h, followed by digestion of remaining DNA templates with 1 μL **DNase I (RNase-free) (Takara, 2270B)** at 37 °C for 1 h. For secondary structure unfolding, 1.5 μg RNA was mixed with 2 μL 20 μM Illumina-SELEX-R primer in a total volume of 10 μL, heated at 70 °C for 5 min, and snap-cooled on ice for 2 min. Reverse transcription was then performed using the **HiScript III 1st Strand cDNA Synthesis Kit (Vazyme, R312-02)** in a 20 μL reaction (10 μL RNA, 2 μL 10 × RT buffer, 2 μL enzyme mix) at 37°C for 1 h 30 min, followed by inactivation at 85 °C for 5 s to generate RNA-DNA hybrids. The RNA strand was then removed using Ribonuclease H (RNase H, Thermo, EN0202) in a 15 μL system (2 μL RNase H, 1.5 μL 10 × reaction buffer) incubated at 37 °C for 45 min. Finally, the ssDNA products were purified using a DNA Clean & Concentrator kit (ZYMO, D4014), eluted in 10 μL nuclease-free water, and used directly for ssDNA-SELEX.

**SELEX**

HT-SELEX and ssDNA-SELEX were performed using identical experimental procedures, as described below. In parallel, an empty-vector control expressing only the Trx-His-FLAG tag scaffold (pET-28a-Trx-His-FLAG) and two human transcription factors, ETV2 (MA0762.1) and HOXB13 (MA0901.1) [6], as positive controls were subjected to the same expression, purification, and SELEX workflow. The empty-vector control was included to assess background nucleic acid recovery arising from the tag scaffold or affinity matrix. 400 ng of protein and 5 µL of DNA input were added to the binding buffer (10 mM Tris pH 7.5, 50 mM NaCl, 1 mM DTT, 1 mM MgCl_2_, 4% glycerol, 0.5 mM EDTA, 5 µg/mL poly-dIdC) to a total volume of 25 µL, and the DNA and protein were incubated at room temperature for 30 minutes. Subsequently, the protein-DNA complex and 10 µL of pre-equilibrated Ni Sepharose 6 Fast Flow resin (GE Healthcare, 17-5318-01) were added to 150 µL of binding buffer. The entire system was transferred to a 96-well plate and incubated at room temperature for 1 h under 800 rpm. After that, the 96-well plate was placed in a washing machine with the washing program set up, using a wash solution (10 mM Tris pH 7.5, 50 mM NaCl, 1 mM DTT, 1 mM MgCl_2_, 4% glycerol, 0.5 mM EDTA) to wash 12 times to remove unbound DNA ligands. Next, the beads were resuspended in 50 μL of ddH_2_O to elute the DNA that preferentially binds to the protein. Finally, the eluted DNA was amplified by PCR using primers listed in Table S3, and the obtained PCR products were used as DNA templates for the next cycle. This process was repeated four times. Each PCR products were purified and sequence using Illumina NovaSeq X Plus PE150.

**EMSA**

Following four rounds of HT-SELEX, the DNA sequences specifically recognized by viral NBPs were converted into seed sequences. These seed sequences were used to scan sequencing data, generating a corresponding PWM. This PWM was then applied to screen the corresponding viral genomes, with a minimum interval of five bases required between adjacent binding sites and a statistical significance threshold set at *p* value < 0.05. The screening results were sorted in ascending order of *p* value, and the top 20 binding sites were selected as potential targets for the viral NBPs. Centered on each binding site, sequences were extended upstream and downstream to a total length of 20 nt. The top three sequences were then selected and synthesized as primers. The reaction mixture was prepared in 5 × EMSA binding buffer, containing 100 pmol of annealed probes and gradient amounts of protein (0–600 pmol) in a total volume of 20 μL. After all components were added, the mixture was gently pipetted up and down to ensure homogeneity. Subsequently, the samples were incubated at room temperature for 1-2 h to facilitate the full binding of proteins to the probes. The reaction mixtures were subsequently analyzed by native polyacrylamide gel electrophoresis. Following electrophoresis, gel bands were visualized using nucleic acid staining dyes, as these bands indicate the formation of protein-nucleic acid complexes.

**qPCR validation of Flag-NBP overexpression**

Overexpression HEK293T cells were transfected with Flag-NBP expression plasmids or empty vector. Total RNA was harvested at 48 h post-transfection, reverse-transcribed into cDNA, and subjected to SYBR Green-based quantitative real-time PCR. Target gene expression was normalized to GAPDH, and relative mRNA abundance was quantified using the method against empty vector controls. All experiments were performed in triplicate, and statistical analysis was performed with unpaired Welch’s *t*-test. The primer sequences used for qPCR are listed in Table S3.

**MitoChIP-qPCR**

MitoChIP-qPCR was performed to validate the physical association of putative NBPs with mtDNA in vivo. Five NBPs predicted to preferentially target mtDNA were randomly chosen from the small-genome DNA virus group (YP_001111256.1, YP_009553535.1, YP_009508841.1, YP_009111420.1, and YP_008431134.1), with their corresponding sequences listed in Table S3. The coding sequences of these NBPs were then inserted into the pcDNA3.1-COX8-3 × Flag (COX8 is a mitochondrial targeting signal peptide.) vector at the NheI and XbaI restriction sites. Homologous recombination was performed using the ClonExpress Ultra One Step Cloning Kit (Vazyme Biotech, C115‑02) following the manufacturer’s instructions. The resulting recombinant plasmids were transiently transfected into HEK293T cells. At 48 h post-transfection, cells were harvested for mitochondrial isolation using a Mitochondria Isolation Kit (MCE, HY-K1060) according to the manufacturer’s instructions, with minor modifications. Briefly, after trypsin digestion and centrifugation, cell pellets were washed with ice-cold PBS, resuspended in mitochondria isolation reagent supplemented with 1 mM PMSF, and incubated on ice for 10-15 min. Cells were then gently homogenized on ice until trypan blue staining indicated ≥ 80% lysis efficiency. The homogenate was centrifuged at 600 g for 10 min at 4 °C to remove nuclei and cell debris, and the supernatant was further centrifuged at 12,000 g for 10 min at 4 °C to pellet crude mitochondria. Mitochondrial pellets were immediately cross-linked with freshly prepared 1% formaldehyde at room temperature for 10 min with gentle rotation. The cross-linking reaction was quenched with 0.125 M glycine for 5 min. After two washes with ice-cold PBS, the cross-linked mitochondria were lysed in RIPA buffer (140 mM NaCl, 1 mM EDTA, 1% Triton X-100, 0.1% SDS, 0.1% sodium deoxycholate, 10 mM Tris-HCl (pH 8.0), and 1 × Proteinase inhibitor (Roche)). Mitochondrial DNA fragmentation was performed using a Covaris sonicator. Covaris M220 sonicator was applied with the following settings: peak power of 75, duty factor of 5%, and cycles/burst of 200, duration for 5 min. After centrifugation at 20,000 g for 10 min at 4 °C, the supernatant was diluted with RIPA buffer, and a 30 μL aliquot was retained as the input sample. Immunoprecipitation was performed using DYKDDDDK affinity beads (Thermo, A36801). Before use, 50 μL of beads per sample was pre-blocked with 1% BSA. The remaining supernatants were incubated with beads at 4 °C overnight with continuous rotation. After incubating, beads were sequentially washed as follows: twice with 1 × RIPA buffer, twice with high-salt RIPA buffer (RIPA plus 0.5 M NaCl), twice with LiCl buffer (250 mM LiCl, 1 mM EDTA, 0.5% IGEPAL CA-630, 0.1% sodium deoxycholate, 10 mM Tris-HCl pH 8.0), and twice with TE buffer (10 mM Tris-HCl pH 8.0, 1 mM EDTA). Each wash step was performed with gentle rocking for 5 min. Protein-DNA complexes were eluted with extraction buffer (1% SDS in 1 × TE). To reverse cross-links, samples were incubated with 5 M NaCl and RNase A (10 mg/mL) at 37 °C for 1 h with shaking, followed by digestion with Proteinase K (10 mg/mL) at 65 °C overnight with thermomixing. DNA was purified using phenol/chloroform/isoamyl alcohol extraction. The abundance of selected mtDNA loci in immunoprecipitated DNA was quantified by real-time quantitative PCR (qPCR) using locus-specific primers in Table S3. MitoChIP-qPCR enrichment was normalized to the input and calculated relative to the empty vector control group.

**MitoChIP-qPCR data normalization**

All MitoChIP‑qPCR signals were normalized using a two‑step strategy: (i) raw Ct values were first normalized to matched input samples; (ii) the input‑normalized values were then further normalized to wild‑type HEK293T signals processed in parallel to remove nonspecific background binding inherent to the cell line. This dual normalization ensures that the final enrichment values specifically reflect NBP‑mtDNA interactions rather than input differences or nonspecific chromatin association.

**Mt/N Ratio quantification for mitochondrial purity and enrichment validation**

To verify mitochondrial purification quality and MitoChIP enrichment efficiency, total DNA was extracted from purified mitochondrial input derived from wild-type HEK293T cells, as well as immunoprecipitated products from YP_001111256.1-mediated MitoChIP. We conducted qPCR to quantify mtDNA and single-copy nuclear DNA levels with primer pairs optimized to avoid amplification of nuclear mitochondrial pseudogenes, and subsequently calculated the mtDNA/nuclear DNA (Mt/N) ratio. All measurements were performed across three independent biological replicates. Statistical analysis was performed with unpaired Welch’s *t*-test (n = 3 biological replicates). The primer sequences used for Mt/N Ratio Quantification are listed in Table S3.

**Mitochondrial membrane potential**

To evaluate the effects of viral proteins on mitochondrial membrane potential, the five aforementioned recombinant NBP-3 × Flag plasmids or empty vector were transfected into HEK293T cells. After 48 h, cells were harvested by trypsinization and resuspended in 0.5 mL complete culture medium. Then, an equal volume of JC-1 (Beyotimes, C2006) staining working solution was added to the cell suspension, followed by gentle mixing and incubation at 5% CO_2_, 37 °C for 20 min in cell culture incubator. During incubation, 1 × JC-1 staining buffer was prepared by diluting the 5 × stock solution with distilled water at a ratio of 1:4 and maintained on ice prior to use. Following staining, cells were centrifuged at 600 g for 3 min at 4 °C, and the supernatant was carefully discarded. Cells were subsequently washed twice with 1 mL 1 × JC-1 staining buffer and centrifuged at 600 g for 3 min at 4 °C. After the final wash, cells were resuspended in 0.5 mL 1 × JC-1 staining buffer and immediately subjected to flow cytometric analysis (Beckman Coulter, Epics XL-MCL). Fluorescence excitation of JC-1 was achieved using a 488 nm argon laser. The results were analyzed using the GraphPad Prism software.

**SELEX data analysis**

Raw sequencing reads were demultiplexed. Only sequences from the 40-bp random region without ambiguous bases (N) were retained for downstream analyses. Autoseed was used to *de novo* discover motifs from the pre-processed SELEX reads **[4,7]** (parameters: -40N < Background sequence > < Signal sequence > 1 8 10 0.35 -50 100), and the corresponding seeds, cycles, and PWMs are provided in **Table S4**. Motif logos were generated using the R package **ggseqlogo [8] (v0.1)**.

**Quantification of enrichment across SELEX cycles**

For each position weight matrix (PWM) of width *W*, all possible *W*-mer DNA sequences were enumerated. The score of a given *W*-mer sequence *S* was calculated using a position-specific log-odds ratio:

$$\text{Score}\left( \text{S} \right)\text{=}\sum_{\text{i}\text{=1}}^{\text{W}} \text{log}_{\text{2}}\left( \frac{\text{P}_{\text{i}}\left( \text{S}_{\text{i}} \right)}{\text{0.25}} \right)$$

where $\text{S}_{\text{i}}$ denotes the nucleotide at position $\text{i}$ of sequence $\text{S}$, and $\text{P}_{\text{i}}\left( \text{S}_{\text{i}} \right)$ denotes the probability of that nucleotide at the corresponding position in the PWM. To exclude low-affinity sequences, only *W*-mers satisfying $\text{P}_{\text{i}}\left( \text{S}_{\text{i}} \right)\text{>0.1}$ at every position and $\text{Score}\left( \text{S} \right)\text{>0}$ were retained.

To remove reverse-complement redundancy, each retained *W*-mer was paired with its reverse complement. The sequence with the higher log-odds score was retained; when the two sequences had identical scores, the originally enumerated sequence was retained. The resulting non-redundant *W*-mers were ranked in descending order according to their log-odds scores to generate the candidate pool.

To control dictionary size and specificity, an empirical false-positive rate (FPR) was estimated using the unselected random 40-nt DNA library from SELEX cycle 0 (C0). The prefiltered, non-redundant *W*-mers were sequentially added to the dictionary from the highest to the lowest score until their cumulative frequency in C0 reached the designated FPR threshold of $\text{10}^{\text{-5}}$. The cumulative frequency was calculated as:

$$\text{Cumulative }\text{f}\text{requency=}\frac{\text{C}_{\text{cum}}}{\text{N}\text{×2×}\left( \text{L}\text{-}\text{W}\text{+1} \right)}$$

where $\text{C}_{\text{cum}}$ is the cumulative match count of the selected entries, $\text{N}$ is the total number of sequencing reads in the C0 library, $\text{L}$ is the length of the randomized region ($\text{L}\text{=40}$), $\text{W}$ is the PWM width, and the factor of 2 represents the two DNA strands.

For each SELEX cycle (C0–C4), k-mer counting was performed using Jellyfish v2.3 with the -C option, which canonicalizes each k-mer and its reverse complement under a single key. The binding frequency of a given *W*-mer dictionary in cycle c was calculated as:

$$\text{Frequency}_{\text{c}}\text{ = }\frac{\text{Match count}_{\text{c}}}{\text{N}_{\text{c}}\text{ × 2 × (}\text{L}\text{ - }\text{W}\text{ + 1)}}$$

where Match count_c_ is the total number of matches to all W-mers in the dictionary, N_c_ is the total number of sequencing reads in cycle C, L is the length of the randomized region (L = 40), W is the PWM width, and the factor of 2 accounts for the two strand orientations included in the search space. Fold enrichment was defined as the ratio of the binding frequency in C4 to that in C0:

$$\text{Fold enrichment = }\frac{\text{Frequency}_{\text{C}\text{4}}}{\text{Frequency}_{\text{C}\text{0}}}$$

To address zero counts for highly specific W-mers in the unselected C0 library, a pseudocount of 1 was added to the C0 match count before calculating the C0 frequency and fold enrichment:

$$\text{Frequency}_{\text{C}\text{0}}\text{ = }\frac{\text{Match count}_{\text{C}\text{0}}\text{ + 1}}{\text{N}_{\text{C}\text{0}}\text{ × 2 × (}\text{L}\text{ - }\text{W}\text{ + 1)}}$$

A motif was considered reliable when it showed clear enrichment relative to cycle 0, progressive enrichment across SELEX rounds, and a stable, interpretable core motif. If no clearly enriched seed was detected, if enrichment did not increase across rounds, if the recovered pattern was dominated by low-complexity, repetitive, or nonspecific sequences, or if no stable core motif could be generated, the protein was considered to have no reliable motif.

**Network Analysis of similarity between PWMs**

**SSTAT was used to** assess pairwise similarity among 33 PWMs [9] (parameters: 50% GC content, pseudocount regularisation, type I threshold = 0.01). A bipartite network was then constructed with two classes of nodes: **viral proteins** and their associated **binding PWMs**. Protein nodes were linked to their corresponding PWMs, which were in turn interconnected if their **SSTAT similarity score** (asymptotic covariance) exceeded **1.0 × 10⁻⁵**, following a previously published SSTAT-based PWM similarity criterion **[3]**. To assess the robustness of module assignments, we additionally applied a more stringent literature-supported cutoff of 1.5 × 10⁻⁵ [5–7]; the major module structure and biological interpretation remained unchanged. All networks were visualized using networkx v2.6.3 and matplotlib v3.5.3.

**Phylogenetics**

**Protein sequences of the 33 viral proteins that yielded successful HT-SELEX experiments were aligned using** **Clustal Omega v1.2.4 with default parameters [10]. Phylogenetic reconstruction was performed with IQ-TREE [11] v3.0.0 under the options -m TEST -B 1000 [12], which enabled automated model selection and ultrafast bootstrap analysis. The best-fit substitution model for each tree was determined using the Bayesian Information Criterion (BIC), resulting in the selection of VT+G4. The phylogenetic trees were visualized with Interactive Tree of Life (iTOL,** <https://itol.embl.de/>**) [13].**

**Structural prediction and similarity analysis**

Protein sequences were uploaded to the **AlphaFold 3** [14] web server to predict pentameric assemblies and protein-DNA complexes using default settings, unless otherwise specified (<https://alphafoldserver.com/>). Local structural confidence was assessed using the predicted Local Distance Difference Test (pLDDT) score, which estimates per-residue prediction confidence on a scale of 0–100. Models with a mean pLDDT > 70 were generally retained for subsequent structural analyses, with the established AlphaFold pLDDT confidence interpretation as a reference [14,15]. YP_008431134.1 (mean pLDDT ≈ 66.5), which fell slightly below this threshold, was additionally retained only for exploratory comparison, and its predicted structural features were interpreted with caution. Structural similarity between protein pairs was quantified using two complementary approaches. The DALI server (Dalilite, version 5) [16] was used to calculate Z-scores, which represent the statistical significance of structural alignments as the number of standard deviations above expected scores for unrelated proteins. TM-align [17] (Version 20220412) was used to calculate TM-scores, a length-normalized metric ranging from 0 to 1 for evaluating global structural similarity, together with root-mean-square deviation (RMSD), which measures the average distance between superimposed Cα atoms. To account for length differences, the average bidirectional TM-scores, normalized by protein length, were calculated. Based on established criteria, A lower RMSD indicates better alignment of the model with the target structure. Following the established DALI criterion, a Z-score > 2 was considered indicative of significant structural similarity, with higher scores indicating greater similarity [16]. Following the established TM-align criterion, TM-scores > 0.5 indicated that two structures share the same fold topology [17], and TM-scores around or below 0.17 are typical of randomly related protein structures **[**17,**18]**. These thresholds were not empirically selected in the present study but were adopted from established criteria described in the original methodological studies. Figures showing structural representations were prepared using PyMOL (Version 3.1, Schrödinger, LLC).

**Prediction and analysis of genomic binding sites**

We scanned 33 PWMs across viral genomes, size-matched host nuclear genomic windows, and host mtDNA using FIMO [19] (v5.5.5) with the parameters --thresh 0.05 --verbosity 1 to identify potential protein-binding sites. For the genome-wide comparison (Figures 1F–1H, S8), only FIMO-predicted sites passing this threshold were retained for downstream analysis. NBPs were grouped according to the size of their cognate viral genomes relative to human mtDNA (~16.6 kb): small-genome viruses, 2.5–7.3 kb (< 0.5-fold of mtDNA); medium-genome viruses, 31.8–37.6 kb (~1.9-to 2.3-fold of mtDNA); and large-genome viruses, 140–212.6 kb (> 8-fold of mtDNA). To enable direct comparison across host and viral genomes, host genomic sequences were partitioned into windows matched to the corresponding viral genome size, and potential NBP-binding sites were identified in these windows using the same FIMO settings. Predicted NBP-binding-site density was calculated as the number of FIMO-predicted sites per kilobase.

To assess whether FIMO-predicted NBP-binding sites were overrepresented in specific genomic contexts, we performed count-based hypergeometric enrichment analyses under a scan-length-normalized null model across four FIMO *p* value thresholds: *p* < 0.05, *p* < 0.01, *p* < 0.005, and *p* < 0.001. For each NBP, FIMO-predicted potential binding sites and scanned sequence lengths were summarized across three genomic contexts: the cognate viral genome, host mtDNA, and host nuclear genome. For viral genomes, the cognate viral genome length was used as the scanned length. Host mtDNA was defined as mtDNA, whereas all non-mitochondrial chromosomes were assigned to the host nuclear genome. Host genomic sequences were partitioned into windows matched to the length of the corresponding viral genome; terminal windows shorter than the viral genome were retained and analyzed using their actual lengths. Each genomic context was tested in a one-versus-rest manner against the other two contexts combined. For each one-versus-rest hypergeometric test, let N denote the total scanned length across the three genomic contexts, K denote the total number of FIMO-predicted potential binding sites across the three contexts, n denote the scanned length of the tested context, and k denote the number of predicted sites observed in that context. Overrepresentation was assessed using the upper-tail hypergeometric probability:

$$\text{P}\text{=}\text{P}\left( \text{x ≥} \text{k} \right)\text{，}\text{w}\text{h}\text{ere}\text{ }\text{X}\text{\textasciitilde}\text{Hypergeometric}\left( \text{N, K, n} \right)$$

Fold enrichment was calculated as:

$$\text{Fold enrichment }\text{=}\frac{\text{observed}}{\text{expected}}\text{=}\frac{\text{kN}}{\text{Kn}}$$

where $\frac{\text{Kn}}{\text{N}}$ represents the expected number of predicted sites in the tested context under a scan-length-normalized null model.

*P* values were adjusted using the Benjamini–Hochberg false discovery rate method, with Holm correction also calculated as a conservative adjustment. Analyses were performed at both per-NBP and group-pooled levels. Per-NBP tests were used to assess heterogeneity among individual NBPs. For the group-pooled analysis, NBPs were first stratified into small-, medium-, and large-genome groups according to the size of their cognate viral genomes. Within each group, predicted site counts and scanned lengths were summed separately for each genomic context. The same one-versus-rest hypergeometric framework was then applied to the pooled counts and pooled scanned lengths. Thus, for a given genome-size group, N represented the sum of scanned lengths across the three genomic contexts for all NBPs in that group, K represented the sum of predicted sites across the three contexts for all NBPs in that group, n represented the summed scanned length of the tested context, and k represented the summed predicted site count in the tested context. The group-pooled tests were used to summarize overall enrichment patterns within each genome-size group. Statistical significance was defined as fold enrichment > 1 and FDR-adjusted *p* < 0.05 (Benjamini–Hochberg correction). Genomic contexts with fold enrichment close to 1 or with non-significant FDR-adjusted *p* values were interpreted as showing no clear enrichment difference.

For the high-resolution analysis of predicted binding-site distributions within the full-length host mtDNA shown in Figures S14A–C, FIMO scanning was performed using a more stringent motif-matching threshold of *p* < 0.005 to retain high-confidence predicted binding sites. Binding-site density was estimated using the kdeplot function from the Python package seaborn (bw_adjust = 0.5), and frequency distributions were visualized using histplot with 100-bp bins.

**Base-label-shuffled PWM analysis**

For each PWM derived from a human-infecting viral NBP, 1,000 matched shuffled PWMs were generated by independently permuting the A/C/G/T probability values within each PWM position, thereby preserving PWM length and the per-position probability distribution while randomizing nucleotide identity. The original and shuffled PWMs were scanned against the same human mtDNA sequence using FIMO, and motif hits were retained at *p* < 0.05, 0.01, 0.005, and 0.001. Fold enrichment was calculated as the observed mtDNA motif-hit count divided by the mean hit count from the shuffled-PWM null distribution. The 95% empirical null interval was defined by the 2.5th and 97.5th percentiles of the null distribution. One-sided empirical *p* values were calculated as (1 + number of shuffled PWMs with hit counts greater than or equal to the observed count)/(1 + 1,000), followed by Benjamini–Hochberg correction separately at each FIMO filtering threshold.

**Dinucleotide-matched nuclear-window null model**

To assess mtDNA motif-hit enrichment, 5,000 mutually non-overlapping 16,569 bp nuclear windows were selected from GRCh38 chromosomes 1–22, X, and Y based on similarity to mtDNA dinucleotide composition. Candidate windows containing > 1% ambiguous bases were excluded, and selection used seed 20260714. The same matched windows were used for all 30 viral PWMs.

FIMO scans were performed on mtDNA and matched windows, and motif hits were counted at *p* < 0.05, 0.01, 0.005, and 0.001. Fold enrichment was calculated as the mtDNA hit count divided by the mean matched-window count. One-sided empirical *p* values were calculated as (1+k)/(1+5000), where k is the number of null windows with counts equal to or greater than the mtDNA count. Benjamini–Hochberg correction was applied across the 30 PWMs separately at each threshold.

**Genome-wide random nuclear-window null model**

A genome-wide random nuclear-window null was used to test whether viral PWM hits were enriched on human mtDNA beyond variation among equal-length nuclear regions. Candidate windows were sampled from the GRCh38 primary assembly chromosomes 1–22, X, and Y, excluding MT. Chromosomes were sampled in proportion to their number of possible 16,569 bp window starts (chromosome length - 16,569 + 1), and start positions were sampled uniformly within chromosomes. Duplicate coordinates were rejected. Coordinates were assigned an original random draw index, grouped by chromosome only for efficient FASTA access, restored to draw order after sequence evaluation, and filtered to retain windows with <= 1% ambiguous bases. Using seed 20260711, the first 10,000 eligible unique windows were retained; overlaps between different selected windows were allowed. The original single-motif PWM for each of 30 viral proteins was scanned with FIMO against real human mtDNA and the random-window FASTA using a maximum reporting threshold of *p* < 0.05. Hits were counted at *p* < 0.05, *p* < 0.01, *p* < 0.005, and *p* < 0.001. Fold enrichment was the observed mtDNA hit count divided by the mean random-window count. One-sided empirical *p* values were calculated as (1 + number of null counts >= observed)/(1 + 10,000), and Benjamini-Hochberg correction was applied across viral PWMs separately within each threshold.

**Prediction of DNA secondary structure at viral protein binding sites**

To predict the secondary structure of viral DNA sequences associated with protein recognition motifs, representative ssDNA fragments were analyzed using the Mfold [20] (UNAFold) web server (https://www.unafold.org/mfold/applications/dna-folding-form.php). For YP_009508805.1, the DNA fragment corresponding to nucleotides 388-404 was extracted from the reference genome NC_039050. This region was selected based on motif scanning results from the FIMO analysis described above, which identified it as a putative viral protein-binding site (*p* value < 0.05). Simulations were performed under conditions approximating human physiological temperature and ionic strength. Specifically, the folding temperature was set to 310.15 K (37 °C), with monovalent and divalent ion concentrations adjusted to 0.1 M Na⁺ and 0.0015 M Mg²⁺ [21,22]. For each sequence, the predicted minimum free energy (ΔG) and melting temperature (Tm) were recorded as measures of thermodynamic stability. Structural features, including the presence of inverted repeats and stem-loop conformations, were annotated based on the predicted folding models. Genomic coordinates corresponding to the analyzed sequences were determined relative to the reference genome.

**Quality assessment of the ssDNA-SELEX input library**

To evaluate the sequence complexity, randomness, and representativeness of the ssDNA-SELEX input library, high-throughput sequencing was performed on the constructed initial ssDNA library used to initiate SELEX. Raw sequencing reads were first processed to extract the designed 40-nt random region. Sequences containing ambiguous bases annotated as “N” were removed from downstream analyses. To avoid biases caused by differences in sequencing depth among samples, valid reads from each sample were randomly down-sampled using a fixed random seed. The down-sampled reads were then pooled, shuffled, and truncated to generate a global dataset containing 6 × 10^6^ valid 40-nt sequences. To quantitatively assess library complexity and sequence randomness, k-mer-based analyses were performed for k values ranging from 2 to 12. This range covers the major length range of the identified PWM motifs in this study. Each 40-nt sequence was decomposed into all overlapping k-mers using a step -1 sliding window, and the occurrence frequency of each k-mer was calculated.

Sequence space coverage was calculated as:

$$\text{Coverage}_{\text{k}}\text{=}\frac{\text{N}_{\text{obs}}\left( \text{k} \right)}{\text{4}^{\text{k}}}\text{*}\text{100\%}$$

Where $\text{N}_{\text{obs}}\left( \text{K} \right)\text{ }$denotes the number of distinct k-mers observed in the library. enotes the number of distinct k-mers observed in the library, and $\text{4}^{\text{k}}$represents the theoretical number of all possible k-mers of length k.

To evaluate the uniformity of k-mer frequency distributions, Shannon entropy was calculated as:

$$\text{H}_{\text{obs}}\text{=-∑}\text{P}_{\text{i}}\text{*}\text{log}_{\text{2}}\left( \text{P}_{\text{i}} \right)\text{}$$

where $\text{P}_{\text{i}}$ denotes the relative frequency of the i-th k-mer, calculated as the count of that k-mer divided by the total number of k-mer occurrences. A higher entropy value indicates a more uniform k-mer distribution and greater sequence randomness. Where:

$$\text{H}_{\max\left( \text{k} \right)}\text{=}\text{log}_{\text{2}}\left( \text{4}^{\text{k}} \right)\text{=2}\text{k}\text{}$$

Thus, $\text{H}_{\text{norm}}\text{=1}$ represents the theoretical maximum randomness.

As a reference, a depth-matched simulated random library was generated in silico by independently sampling each nucleotide position from a uniform distribution of A, C, G, and T, with a probability of 0.25 for each base. The simulated random library was processed using the same k-mer analysis pipeline as the observed ssDNA-SELEX input library. Sequence space coverage and normalized Shannon entropy were then compared between the observed library and the simulated random reference to evaluate the complexity, representativeness, and randomness of the ssDNA-SELEX input library.

**Classification of PWM binding patterns as non-repetitive (monomer-like) or containing two similar subsequences (dimer-like)**

Each SELEX-derived motif was represented as a 4 × n matrix of A/C/G/T counts and normalized column-wise without pseudocounts to obtain a PWM of base probabilities. For each PWM, all pairs of equal-length, contiguous, and non-overlapping subsequences were examined. subsequence length L was restricted to:

$$\text{4≤}\text{L}\text{≤}\left\lfloor\text{n}/\text{2} \right\rfloor$$

For each subsequence pair, the second subsequence was compared with the first under four transformations: direct, complement, reverse, and reverse complement. A single transformation was applied consistently across the entire subsequence. The expected positional base-match fraction was calculated as:

$$\text{M}\left( \text{B}_{\text{1}}\text{,}\text{B}_{\text{2}}\text{,}\text{T} \right)\text{=}\frac{\text{1}}{\text{L}}\sum_{\text{i}\text{=1}}^{\text{L}} \sum_{\text{b}\text{∈}\left\{ \text{A}\text{,}\text{C}\text{,}\text{G}\text{,}\text{T} \right\}} \text{P}_{\text{B}_{\text{1}}\text{,}\text{i}}\left( \text{b} \right)\text{ }\text{P}_{\text{T}\left( \text{B}_{\text{2}} \right)\text{,}\text{i}}\left( \text{b} \right)$$

where $\text{P}\left( \text{B}^{\text{1}}\text{,}\text{i}\text{,}\text{b} \right)$ and $\text{P}\left( \text{T}\left( \text{B}^{\text{2}} \right)\text{,}\text{i}\text{,}\text{b} \right)$ denote the probabilities of base b at position i in the first subsequence and the transformed second subsequence, respectively. The observed statistic M^*^ was defined as the maximum score across all eligible subsequence lengths, subsequence positions, subsequence pairs, and transformations:

$$\text{M}^{\text{*}}\text{=}\max_{\text{L}\text{,}\text{B}_{\text{1}}\text{,}\text{B}_{\text{2}}\text{,}\text{T}} \text{M}\left( \text{B}_{\text{1}}\text{,}\text{B}_{\text{2}}\text{,}\text{T} \right)$$

Statistical significance was assessed separately for each PWM using 10,000 complete-column permutations. Each permutation preserved the complete probability vector of every PWM column while disrupting its positional arrangement. The full subsequence-pair scan was repeated for each permuted PWM to generate a null distribution of M^*^. The Monte Carlo *p* value was calculated as:

$$\text{p}\text{=}\frac{\text{1+}\sum_{\text{r}\text{=1}}^{\text{10000}} \text{I}\left( \text{M}_{\text{null}\text{,}\text{r}}^{\text{*}}\text{≥}\text{M}_{\text{observed}}^{\text{*}} \right)}{\text{100}\text{01}}$$

The *p* values from all 33 PWMs were adjusted together using the Benjamini–Hochberg procedure. PWMs with qBH < 0.05 were operationally classified as **binding patterns containing two similar subsequences (dimer-like)**, whereas the remaining PWMs were classified as **non-repetitive binding patterns (monomer-like), indicating that no significant similarity between two subsequences was detected**. These classifications describe the sequence organization of the PWMs rather than the physical oligomeric state of the corresponding proteins. Analyses were performed using NumPy 2.0.2 with a fixed random seed of 20260715.

**Statistical analysis**

Statistical details for the following experiments are provided: (i) MitoChIP-qPCR validation and (ii) mitochondrial membrane potential analysis by JC-1 staining. All experiments were performed with three independent biological replicates, and data are presented as mean ± standard deviation (SD). Before parametric comparisons, the normality of each group was assessed using the Shapiro-Wilk test, and homogeneity of variances was examined with the Brown-Forsythe test.

For JC-1 staining, the Brown-Forsythe test indicated equal variances across groups; therefore, ordinary one-way ANOVA was applied, followed by Dunnett's post-hoc test for comparisons between each experimental group and the empty vector, correcting for multiple comparisons.

For MitoChIP-qPCR, the Brown-Forsythe test revealed unequal variances; accordingly, Welch one-way ANOVA, which does not assume homogeneity of variances, was employed. Pairwise comparisons against the empty vector were performed using Dunnett's T3 post-hoc test, which is robust to variance heterogeneity and also controls for multiplicity.

For both assays, exact *p* values are reported on figure and effect sizes (mean difference, Δ), and 95% confidence intervals are reported directly on the Table S14 and Table S17.

**References**

1. Brister, J. Rodney, Danso Ako-Adjei, Yiming Bao, Olga Blinkova. 2015. “NCBI viral genomes resource.” *Nucleic Acids Research* 43: D571–D577. <https://doi.org/10.1093/nar/gku1207>

2. Mihara, Tomoko, Yosuke Nishimura, Yugo Shimizu, Hiroki Nishiyama, Genki Yoshikawa, Hideya Uehara, Pascal Hingamp, Susumu Goto, Hiroyuki Ogata. 2016. “Linking virus genomes with host taxonomy.” *Viruses* 8: 66. <https://doi.org/10.3390/v8030066>

3. Fan, Shaorong, Wenju Sun, Ligang Fan, Nan Wu, Wei Sun, Haiqian Ma, Siyuan Chen, et al. 2022. “The highly conserved RNA-binding specificity of nucleocapsid protein facilitates the identification of drugs with broad anti-coronavirus activity.” *Computational and Structural Biotechnology Journal* 20: 5040–5044. <https://doi.org/10.1016/j.csbj.2022.09.007>

4. Li, Tian, Hao Chen, Nana Ma, Dingkun Jiang, Jiacheng Wu, Xinfeng Zhang, Hao Li, et al. 2025. “Specificity landscapes of 40 R2R3‐MYBs reveal how paralogs target different cis‐elements by homodimeric binding.” *iMeta* 4: e70009. <https://doi.org/10.1002/imt2.70009>

5. Fan, Ligang, Tingting Wang, Canfeng Hua, Wenju Sun, Xiaoyu Li, Lucas Grunwald, Jingui Liu, et l. 2020. “A compendium of DNA-binding specificities of transcription factors in Pseudomonas syringae.” *Nature Communications* 11: 4947. <https://doi.org/10.1038/s41467-020-18744-7>

6. Jolma, Arttu, Jian Yan, Thomas Whitington, Jarkko Toivonen, Kazuhiro R. Nitta, Pasi Rastas, Ekaterina Morgunova, et al. 2013. “DNA-binding specificities of human transcription factors.” *Cell* 152: 327–339. <https://doi.org/10.1016/j.cell.2012.12.009>

7. Nitta, Kazuhiro R, Arttu Jolma, Yimeng Yin, Ekaterina Morgunova, Teemu Kivioja, Junaid Akhtar, Korneel Hens, et al. 2015. “Conservation of transcription factor binding specificities across 600 million years of bilateria evolution.” *Elife* 4: e04837. <https://doi.org/10.7554/eLife.04837>

8. Wagih, Omar. 2017. “ggseqlogo: a versatile R package for drawing sequence logos.” *Bioinformatics* 33: 3645–3647. <https://doi.org/10.1093/bioinformatics/btx469>

9. Pape, Utz J, Sven Rahmann, Martin Vingron. 2008. “Natural similarity measures between position frequency matrices with an application to clustering.” *Bioinformatics* 24: 350–357. <https://doi.org/10.1093/bioinformatics/btm610>

10. Sievers, [Fabian](https://onlinelibrary.wiley.com/authored-by/Sievers/Fabian), [Desmond](https://onlinelibrary.wiley.com/authored-by/Higgins/Desmond+G.) G. Higgins. 2018. “Clustal Omega for making accurate alignments of many protein sequences.” *Protein Science* 27: 135–145. <https://doi.org/10.1002/pro.3290>

11. Nguyen, Lam-Tung, Heiko A. Schmidt, Arndt Haeseler, Bui Quang Minh. 2015. “IQ-TREE: a fast and effective stochastic algorithm for estimating maximum-likelihood phylogenies.” Molecular Biology and Evolution 32: 268–274. <https://doi.org/10.1093/molbev/msu300>

12. Nomburg, Jason, Erin E. Doherty, Nathan Price, Daniel Bellieny-Rabelo, Yong K. Zhu, Jennifer A. Doudna. 2024. “Birth of protein folds and functions in the virome.” *Nature* 633: 710–717. <https://doi.org/10.1038/s41586-024-07809-y>

13. Letunic, Ivica, Peer Bork. 2021. “Interactive Tree Of Life (iTOL) v5: an online tool for phylogenetic tree display and annotation.” *Nucleic Acids Research* 49: W293–W296. <https://doi.org/10.1093/nar/gkab301>

14. Abramson, Josh, Jonas Adler, Jack Dunger, Richard Evans, Tim Green, Tim Pritzel, Olaf Ronneberger, et al. 2024. “Accurate structure prediction of biomolecular interactions with AlphaFold 3.” *Nature* 630: 493–500. <https://doi.org/10.1038/s41586-024-07487-w>

15. Jumper, John, Richard Evans, Alexander Pritzel, Tim Green, Michael Figurnov, Olaf Ronneberger, Kathryn Tunyasuvunakool, et al. 2021. “Highly accurate protein structure prediction with AlphaFold.” *Nature* 596: 583–589. <https://doi.org/10.1038/s41586-021-03819-2>

16. Holm, Liisa. 2022. “Dali server: structural unification of protein families.” *Nucleic Acids Research* 50: W210–W215. <https://doi.org/10.1093/nar/gkac387>

17. Zhang, Yang, Jeffrey Skolnick. 2005. “TM-align: a protein structure alignment algorithm based on the TM-score.” *Nucleic Acids Research* 33: 2302–2309. <https://doi.org/10.1093/nar/gki524>

18. Xu, Jinrui, Yang Zhang. 2010. “How significant is a protein structure similarity with TM-score= 0.5?” *Bioinformatics* 26: 889–895. <https://doi.org/10.1093/bioinformatics/btq066>

19. Grant, Charles E., Timothy L. Bailey, William Stafford Noble. 2011. “FIMO: scanning for occurrences of a given motif.” *Bioinformatics* 27: 1017–1018. <https://doi.org/10.1093/bioinformatics/btr064>

20. Zuker, Michael. 2003. “Mfold web server for nucleic acid folding and hybridization prediction.” *Nucleic Acids Research* 31: 3406–3415. <https://doi.org/10.1093/nar/gkg595>

21. Owczarzy, Richard, Bernardo G. Moreira, Yong You, Mark A. Behlke, Joseph A. Walder. 2008. “Predicting stability of DNA duplexes in solutions containing magnesium and monovalent cations.” *Biochemistry* 47: 5336–5353. <https://doi.org/10.1021/bi702363u>

22. SantaLucia Jr, John. 1998. “A unified view of polymer, dumbbell, and oligonucleotide DNA nearest-neighbor thermodynamics.” *Proceedings of the National Academy of Sciences of the United States of America (PNAS)* 95: 1460–1465. <https://doi.org/10.1073/pnas.95.4.1460>

**Supplementary figures**

**Figure S1** **SDS-PAGE and anti-FLAG Western blot analysis of all 106 purified viral candidate NBPs.** (A–C) Proteins were expressed in *E. coli* as dual-tagged constructs (His-tag + FLAG-tag), purified by Ni^2+^-affinity chromatography via the His-tag, and visualized by Coomassie‑stained SDS-PAGE (A and B) or anti-FLAG Western blotting (C). For proteins with sufficient yield, SDS-PAGE was used to assess purity and molecular weight; for low-abundance proteins, anti-FLAG Western blotting was applied to confirm successful. FT is the abbreviation for Flow-through.

**Figure S2 Positive and background controls for the HT-SELEX workflow. (A**) Sequence logos compare HT-SELEX-derived motifs with corresponding JASPAR reference motifs for HOXB13 and ETV2 [6]. Values in parentheses indicate JASPAR matrix IDs. Motifs recovered from two independent HT-SELEX replicates were consistent with the JASPAR reference motifs at the core positions. The empty-vector/tag-only control was processed using the same workflow but did not yield a clear motif. N/A indicates that no clear motif was detected. **(B**) PWM similarity networks for HOXB13 and ETV2 positive controls. Diamonds represent individual PWM models, and circles represent the corresponding protein entries. Protein nodes are connected to their associated PWM models, and PWM models are connected to each other when their SSTAT similarity score, measured as asymptotic covariance, is greater than 1.0 × 10⁻⁵. The close connections among JASPAR and replicate-derived PWMs support the reproducibility of the HT-SELEX-derived motifs.

**Figure S3 Round-by-round enrichment of motif-containing sequences during HT-SELEX.** Each panel represents one viral nucleic acid-binding protein, labeled by its protein accession number. The x-axis shows the SELEX cycle, with **C0** representing the initial random dsDNA library and **C1–C4** representing successive selection cycles. The y-axis shows the normalized enrichment level of motif-containing reads in each cycle, calculated as the fraction of motif-containing reads in a given cycle divided by the corresponding fraction in C0. Motif-containing reads were identified using a motif-matching threshold corresponding to **FPR = 1 × 10⁻⁵**. An enrichment level of 1 indicates no enrichment relative to the random input library, whereas values greater than 1 indicate increased representation of motif-containing sequences during selection.

**Figure S4 Motif reproducibility between replicate HT-SELEX experiments.** Sequence logos show DNA-binding motifs obtained for 16 viral proteins, each profiled in two replicate HT-SELEX experiments. For each protein, Rep1 and Rep2 indicate the two replicate experiments. PWM similarity between replicates was quantified using SSTAT, with the corresponding Ssum similarity score (asymptotic covariance) shown above each motif pair; Ssum values > 1.0 × 10⁻⁵ were considered indicative of motif similarity. All replicate pairs retained highly similar core recognition sequences, supporting the reproducibility of HT-SELEX motif discovery.

**Figure S5** **EMSA verification of SELEX-identified DNA binding motifs for viral NBPs.** Gradient concentrations of each candidate viral NBP were incubated with 100 pmol wild-type motif-containing DNA probe for EMSA detection. “+” and “−” indicate addition or omission of DNA probe, respectively, and motif sequence logos are shown above each gel. Two representative proteins were further tested with wild-type (WT) and motif-mutated (Mut) probes (M = DNA ladder). The core bases highlighted in red boxes in the wild-type motif were replaced with nucleotides marked in blue boxes in the mutant probes. Loss of specific band shifts upon motif mutagenesis confirms sequence-specific binding between viral NBPs and their predicted target DNA motifs.

**Figure S6 Network of similarity among the obtained PWMs from viral proteins.** (A) **Heatmap showing pairwise within-module SSTAT similarity scores for modules containing more than one viral protein. Cell values are shown as similarity score × 10⁻⁵; only pairs with similarity score ≥ 1.0 × 10⁻⁵ are displayed. Protein IDs are colored by module membership, and black grid lines separate modules 1–5. Blue-outlined cells indicate borderline pairs with 1.0 × 10⁻⁵ ≤ similarity score < 1.5 × 10⁻⁵. Using similarity score** > **1.5 × 10⁻⁵ as the more stringent cutoff, all multi-member modules retained within-module links. Relaxing the cutoff to similarity score ≥ 1.0 × 10⁻⁵ introduced only four additional links, all within module 1, and no between-module links, indicating that the major module structure was stable across the tested similarity cutoffs.** (B) Viral protein similarity network with motif logos, related to Figure 1C. The network consists of 21 distinct modules of viral NBPs corresponding to the obtained PWMs. Diamonds denote individual PWM models; circles denote viral proteins; three NBPs from non-human-infecting viruses (YP_010782993.1, NP_671816.1, AVM80381.1) were included as non-human-virus controls. Circle fill colors indicate the viral families from which the proteins originate. Dashed annotations label the protein IDs of viral proteins. An edge is shown between two nodes if the SSTAT similarity score > 1 × 10⁻⁵; edge colors reflect the SSTAT score. For additional details, see Table S4 and Table S5.

**Figure S7** **Structural analysis of CCACC motif viral NBPs in module 1.** (A–I) Side and top views of AlphaFold 3-predicted pentameric assemblies of the nine viral NBPs in module 1 that recognize the CCACC motif. The mean pLDDT score of each model is indicated in parentheses. One subunit in each pentamer is highlighted in green for clarity. pLDDT reflects per-residue local prediction confidence, with higher values indicating greater confidence in the predicted local structure. (J) Distribution of the mean pLDDT scores of the seven models retained for structural analysis. Models with markedly lower confidence scores were excluded, whereas YP_008431134.1 (mean pLDDT = 66.5) was retained as a borderline case for exploratory comparison. (K–M) Pairwise structural comparisons among the high-confidence AlphaFold3-predicted module 1 structures and the experimentally determined crystal structure (PDB: 4MJ1). Circle fill colors indicate the viral families from which the proteins originate; diagonal self-comparisons were omitted. (K) Pairwise DALI Z-score heatmap. Structural similarities between the A chains of each structure were calculated using DaliLite v5. Higher Z-scores indicate greater structural similarity; Z-scores > 2 were considered significant according to established DALI criteria. NA denotes pairs not reported as significant by DaliLite. (L) Pairwise RMSD heatmap. Structural alignments of the pentameric assemblies were performed using TM-align. Each cell shows the root-mean-square deviation (RMSD) between superimposed Cα atoms, with lower RMSD values indicating closer structural agreement. (M) Pairwise TM-score heatmap. Structural similarity of the pentameric assemblies was evaluated using TM-align. Each cell shows TM_score_1, TM_score_2, and TM_score_avg from top to bottom. TM_score_1 and TM_score_2 are normalized by the lengths of the first/query and second/target structures, respectively, whereas TM_score_avg is their mean and was used as the summary measure. Following the established TM-align criterion, TM-scores > 0.5 were considered indicative of a similar overall fold. Further details are provided in the Methods and Table S7.

**Figure S8 Three-genomic-context hypergeometric enrichment analysis of predicted NBP-binding sites.** NBPs were grouped by the size of their cognate viral genomes relative to human mtDNA (~16.6 kb): large, 140–212.6 kb (> 8-fold of mtDNA); medium, 31.8-37.6 kb (~1.9-to 2.3-fold of mtDNA); and small, 2.5–7.3 kb (< 0.5-fold). (A) **Group-pooled enrichment analysis across the three genomic contexts at multiple FIMO thresholds.** For each genome-size group, FIMO-predicted potential NBP-binding-site counts and scanned sequence lengths were summed within each genomic context before enrichment testing. Fold enrichment was calculated using the scan-length-based one-versus-rest hypergeometric framework for the cognate viral genome, human mtDNA, and human nuclear genome at FIMO *p* value thresholds of *p* < 0.05, *p* < 0.01, *p* < 0.005, and *p* < 0.001. The dashed horizontal line indicates no enrichment (fold enrichment = 1). Values above the bars indicate fold enrichment. The number of NBPs included in each genome-size group is shown below the group labels. Statistical significance was determined using Benjamini–Hochberg-adjusted *p* values; ***, FDR-adjusted *p* < 0.001; ns, not significant. (B) Per-protein enrichment analysis across all NBPs. Enrichment was calculated individually for each NBP, and NBPs are grouped by viral genome size for display. Protein IDs highlighted in red indicate module-1 NBPs.

**Figure S9 Empirical nuclear-window null analyses of mtDNA motif-hit enrichment.** For each PWM derived from a human-infecting viral NBP, motif hits on human mtDNA were compared with hits in two sets of 10,000 nuclear genomic windows of the same length as mtDNA. (A) Dinucleotide-matched nuclear windows were selected from 1,000,000 randomly sampled windows from chromosomes 1–22, X, and Y of GRCh38 according to their similarity to mtDNA in GC and CpG content. (B) Nuclear windows were randomly sampled from the same chromosomes without composition matching. Human mtDNA and the nuclear windows were scanned using identical FIMO parameters, and motif hits were retained at four *p* value filtering thresholds (*p* < 0.05, 0.01, 0.005, and 0.001; left to right). Fold enrichment was calculated as the observed mtDNA motif-hit count divided by the mean hit count across the corresponding nuclear-window null distribution. In the genome-size-group panels, individual points represent proteins, black horizontal bars indicate group medians, and the annotated values show the corresponding median fold enrichments. In the per-protein panels, points show fold-enrichment estimates and horizontal lines indicate the 95% empirical null intervals, defined by the 2.5th and 97.5th percentiles of the window-level hit-count distribution and normalized to the null mean. Dashed lines indicate no enrichment (fold enrichment = 1). Viral NBPs are grouped as Large (blue, n = 8), Medium (orange, n = 7), or Small (green, n = 15). The seven small-genome module 1 NBPs are highlighted as red diamonds, with their corresponding empirical null intervals shown in red.

**Figure S10 mtDNA motif-hit enrichment relative to shuffled-PWM controls.** For each of 30 human-infecting viral NBPs, 1,000 matched control PWMs were generated by independently permuting the A/C/G/T probability labels within each PWM position, thereby preserving PWM length and the per-position probability-value set while randomizing nucleotide identity. The original and shuffled PWMs were scanned against the same human mitochondrial genome using identical parameters, and motif hits were evaluated at four FIMO *p* value filtering thresholds *(p* < 0.05, 0.01, 0.005, and 0.001; left to right). Fold enrichment was calculated as the mtDNA motif-hit count obtained with the original PWM divided by the mean hit count from the corresponding shuffled-PWM null distribution. The upper row summarizes fold enrichment by viral genome-size group. Individual markers represent proteins, black horizontal bars indicate group medians, and the values above each group report the corresponding median fold enrichment. The lower row shows per-protein fold-enrichment estimates. Horizontal segments indicate the central 95% empirical null interval, defined by the 2.5th and 97.5th percentiles of the shuffled-PWM hit-count distribution and normalized to the corresponding null mean. These intervals are null reference ranges rather than confidence intervals for the observed fold enrichment. Blue, orange, and green circles denote non-Module 1 proteins from the large-, medium-, and small-genome groups, respectively, whereas red diamonds denote Module 1 proteins. Filled markers indicate significant enrichment based on one-sided empirical *p* values with Benjamini–Hochberg-adjusted *p* value< 0.05, whereas open markers indicate no significant enrichment. Dashed reference lines denote fold enrichment = 1, shown horizontally in the upper row and vertically in the lower row. Benjamini–Hochberg correction was applied across the 30 proteins separately at each FIMO filtering threshold.

**Figure S11 Genome-wide binding analysis of non-human-infecting viral proteins.** Genome-wide binding analysis of viral proteins YP_010782993.1 (rat host) and NP_671816.1 (Marmota monax host) from non-human-infecting viruses across their corresponding viral genomes, host genomes, and host mtDNA (with host data windowed to match each viral genome size). The x-axis shows viral protein IDs, the left y-axis shows predicted target counts per kilobase (TCPK), and the right y-axis shows viral genome size. Green, black, and red circles indicate the mean predicted TCPK on human mtDNA, human nuclear chromosomes, and cognate viral genomes, respectively. Human mtDNA and nuclear chromosomes were analyzed using windows matched to each viral genome size. Blue diamonds indicate viral genome size.

 **Figure S12** **Genome-wide distribution of predicted binding sites for viral protein in module 1 across viral genomes.** Frequency histograms (blue bars) and density curves (red lines) show the genome-wide distributions of predicted binding sites for each viral protein. Each panel corresponds to a viral protein, with the protein accession number and genome length indicated in parentheses. The x-axis represents viral genome coordinates (in kilobases), with each histogram bin representing a 100 bp window. The left y-axis shows the frequency of predicted target binding sites per 100 bp, while the right y-axis shows the density of these motifs. Discrete peaks represent localized regions of high binding-site density, suggesting potential sequence hotspots that may serve as nucleation sites for viral genome assembly or packaging.

**Figure S13 Genome-wide distribution of predicted binding sites for viral proteins from modules other than module 1 across their cognate viral genomes.** Frequency histograms (blue bars) and density curves (red lines) show the genome-wide distributions of predicted binding sites for each viral protein. Each panel corresponds to a viral protein, with the protein accession number and genome length indicated in parentheses. The x-axis represents viral genome coordinates (in kilobases), with each histogram bin representing a 100 bp window. The left y-axis shows the frequency of predicted target binding sites per 100 bp, while the right y-axis shows the density of these motifs. Discrete peaks represent localized regions of high binding-site density, suggesting potential sequence hotspots that may serve as nucleation sites for viral genome assembly or packaging.

**Figure S14 Predicted binding landscape of module 1 viral NBPs from human-infecting viruses across human mtDNA.** (A) Binding site density across the full human mtDNA. Red curve: Binding density on mtDNA for proteins in module 1 from human-infecting viruses. Gray curve: Binding density on mtDNA for non-module-1 proteins from human-infecting viruses. (B) MtDNA annotations and viral proteins in module 1 from human-infecting viruses binding landscape. Top: Linear map of the human mtDNA (16,569 bp) with feature classes: D-loop (purple), protein-coding (blue), rRNA (orange), tRNA (magenta), and other annotations (gray). Gene annotations are shown above the track, with the MT prefix omitted. Bottom: Stacked frequency histograms of predicted binding sites for Module 1 viral proteins from human-host viruses along mtDNA (bin = 200 bp). Bars show the per-bin site counts, stacked by viral protein (colors keyed in the legend). x-axis: mtDNA genome coordinates (kb). y-axis: Stacked count (200 bp per bin). Calculation details and motif thresholds are provided in the Methods. (C) Heatmap of predicted target counts per kilobase (TCPK) across mitochondrial genes for the viral proteins in module 1 from human-infecting viruses, ranked by TCPK per gene in descending order. For additional details, see Table S13.

**Figure S15** **Validation of Flag-NBP overexpression efficiency by quantitative PCR.** Quantitative PCR was performed to assess the mRNA-level overexpression efficiency of all Flag-tagged NBP constructs, with GAPDH serving as the endogenous reference gene for normalization. For each individual construct, relative fold enrichment of target mRNA was calculated against its corresponding empty vector control from the same experimental batch. All candidate NBP proteins and the negative control construct exhibited robust overexpression relative to their matched empty vector controls, which verified sample quality and validated the reliability of downstream immunoprecipitation assays. Data are shown as mean ± SD, and p values were calculated by unpaired *t*-test (with Welch's correction). It should be noted that these statistical comparisons serve exclusively as quality control (QC) to verify overexpression efficiency. Thus, corrections for multiple comparisons were not applied, and these results should not be interpreted as primary statistical evidence for the biological conclusions.

**Figure S16** **Mt/N ratio verifies mitochondrial purity and MitoChIP enrichment efficiency.** Mt/N (mtDNA/nuclear DNA) ratios were quantified by qPCR in mitochondrial input fractions from wildtype HEK293T cells and MitoChIP-enriched samples from YP_001111256.1 overexpressing HEK293T cells. Assays used a validated protocol to avoid pseudogene co-amplification. The statistical comparison presented here serves exclusively as quality control (QC) to confirm mitochondrial enrichment and consistency. Therefore, while the *p* value is reported, these findings are intended strictly as QC validation and should not be considered primary statistical evidence supporting the biological conclusions of this study. Statistics were performed with Welch’s *t*-test; data are mean ± SD. The YP_001111256.1 ChIP group showed a significantly higher Mt/N ratio than the input group (*p* = 0.0041, t = 15.48, df = 2.004, 95% CI 735.9–1301).

**Figure S17 Quality assessment and validation of the ssDNA‑SELEX workflow.** (A) Schematic overview of the ssDNA library generation strategy for ssDNA-SELEX. Color coding indicates defined sequence elements throughout the workflow, including primer regions, the 40-nt randomized region, the T7 promoter, and the final Illumina Tru-seq adapter-compatible architecture. Primer sequences are listed in Table S3. (B) Agarose gel electrophoresis analysis of key intermediate products during ssDNA-SELEX library construction. Agarose gel (2.5%) electrophoresis showing the library products at different preparation stages. From left to right: DNA marker (with size labels in base pairs, bp), primary double-stranded DNA (dsDNA) library, T7 promoter-ligated dsDNA library, in vitro transcribed complementary DNA (cDNA) library, and the final single-stranded DNA (ssDNA) library. The gel demonstrates the successful progression of each step, with the expected size shifts: the primary dsDNA library (176 bp), the T7-ligated library (196 bp, after promoter addition), the shorter cDNA transcript (179 bp), and the final purified ssDNA library (179 nt). (C) K-mer space coverage of the observed ssDNA-SELEX input library and a depth-matched simulated random reference across k = 2 to k = 12. Coverage was calculated as the percentage of distinct observed k-mers among all theoretically possible 4^k^ k-mers. The k range of 2–12 was selected because it covers the major length range of the identified motifs in this study. (D) Normalized Shannon entropy of the k-mer frequency distribution across k = 2 to k = 12. The observed library showed normalized entropy values close to 1 and comparable to those of the simulated random reference, indicating high sequence randomness and minimal k-mer distribution bias. The k range of 2–12 was selected because it covers the major length range of the identified motifs in this study. (E) Positive-control ssDNA-SELEX experiments using protein with known and experimentally validated ssDNA-binding activity (translocated in liposarcoma/fused in sarcoma, FUS/TLS). Sequence logos from replicate experiments (Rep1 and Rep2) show enriched motifs. Core recognition sequences are highlighted in red boxes. Reproducibility across replicates confirms that the ssDNA-SELEX workflow reliably captures the ssDNA-binding specificity of this positive-control protein.

**Figure S18 Viral NBP PWMs classified as dimer-like binding patterns containing two similar subsequences.** Sequence logos show the six PWMs classified as dimer-like based on 10,000 complete-column permutations followed by Benjamini–Hochberg correction (q_BH_ < 0.05). The two equal-length, non-overlapping subsequences are highlighted in blue and red. Panel headings indicate the Protein ID, q_BH_ and M^∗^, the maximum expected positional base-match fraction. The dimer classification depends only on the statistical significance of M^*^ (q_BH_ < 0.05). Complete classification results for all 33 PWMs are provided in Table S19.
